# Targeting LIF With Cyclovirobuxine D to Suppress Tumor Progression via LIF/p38MAPK/p62‐Modulated Mitophagy in Hepatocellular Carcinoma

**DOI:** 10.1002/mco2.70227

**Published:** 2025-05-24

**Authors:** Yingying Shao, Di Lu, Wenke Jin, Sibao Chen, Lifeng Han, Tao Wang, Leilei Fu, Haiyang Yu

**Affiliations:** ^1^ State Key Laboratory of Chinese Medicine Modernization Tianjin University of Traditional Chinese Medicine Tianjin China; ^2^ School of Medicine Nankai University Tianjin China; ^3^ Affiliated Hangzhou First People's Hospital Zhejiang University School of Medicine Hangzhou China; ^4^ Department of Biotherapy Cancer Center and State Key Laboratory of Biotherapy West China Hospital Sichuan University Chengdu China; ^5^ Sichuan Engineering Research Center for Biomimetic Synthesis of Natural Drugs School of Life Science and Engineering Southwest Jiaotong University Chengdu China; ^6^ State Key Laboratory of Chinese Medicine and Molecular Pharmacology (Incubation) Shenzhen, Department of Applied Biology and Chemical Technology Research Center for Chinese Medicine Innovation The Hong Kong Polytechnic University Hong Kong China; ^7^ Haihe Laboratory of Modern Chinese Medicine Tianjin China

**Keywords:** cyclovirobuxine D (CVB‐D), hepatocellular carcinoma (HCC), leukemia inhibitory factor (LIF), mitophagy, small‐molecule inhibitor

## Abstract

Leukemia inhibitory factor (LIF) exerts an oncogenic function in several types of cancer, including hepatocellular carcinoma (HCC). However, small‐molecule inhibitors of LIF haven't been established. Here, we identified that LIF was remarkably overexpressed in HCC by multi‐omics approaches, indicating that inhibition of LIF would be a promising therapeutic strategy. Inhibiting LIF could suppress proliferation and metastasis by activating p38MAPK/p62‐modulated mitophagy. Interestingly, we found that the natural small‐molecule Cyclovirobuxine‐D (CVB‐D), was a new inhibitor of cytoplasmic LIF in HCC. We further validated LIF as a potential target of CVB‐D through biotin‐modified CVB‐D‐Probe utilizing mass spectrometry. Mechanistically, we showed that CVB‐D could bind to LIF at Val145, thereby inducing mitophagy, accompanied by cell cycle arrest and inhibition of invasion and migration. Moreover, we demonstrated that CVB‐D had a therapeutic potential by targeting LIF‐modulated mitophagy in patient‐derived xenograft (PDX) models, which would elucidate LIF as a druggable target and regulatory mechanisms and exploit CVB‐D as the novel small‐molecule inhibitor of LIF for future HCC drug discovery.

## Graphical abstract

Multi‐omics approaches identify LIF as a potential biomarker and druggable protein that is dysregulated in HCC. LIF inhibition suppresses HCC development by inducing mitophagy via the novel LIF/p38MAPK/p62 pathway. Cyclovirobuxine‐D (CVB‐D) was identified as the first LIF inhibitor in HCC, which binding LIF at Val145, showed therapeutic potential in both nude mouse and patient‐derived xenograft (PDX) models by inducing mitophagy, cell cycle arrest, suggesting a therapeutic potential drug for future cancer treatment.

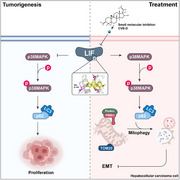

## 1 Introduction

Hepatocellular carcinoma (HCC) ranks as the sixth most lethal cancer globally among all malignancies [1]. Sorafenib and lenvatinib, as multi‐kinase inhibitors, have secured approval as first‐line clinical treatments for HCC. In addition, nivolumab, regorafenib, and cabozantinib have been approved for clinical use in HCC therapy [2, 3, 4]. However, owing to the high recurrence rate of HCC, these drugs only marginally extend the median survival by a few months. Moreover, high rates of metastasis of HCC render poor prognosis, with limited druggable targets and therapeutic strategies that have been exploited to effectively address this issue [5]. Thus, the lack of effective targeted therapies and unvalidated oncogenic mechanisms urgently necessitates the uncovering of new druggable targets and novel small‐molecule drugs to improve HCC therapy.

Leukemia inhibitory factor (LIF), an intricate member of the Interleukin‐6 (IL‐6) cytokine family, exerts pleiotropic effects, intricately regulating cell proliferation and immune response [6, 7, 8]. The alternative splicing of the *lif* gene produces three transcripts, LIF‐D (secreted), LIF‐M (secreted and intracellular), and LIF‐T (intracellular), and expressed in a highly tissue and cell‐type‐dependent manner [9, 10]. Importantly, LIF e exerts a complex influence on cancer development, exhibiting both tumor‐suppressive effects (mainly in leukemia) and tumor‐promoting effects (in many solid tumors, including pancreatic, breast, and colorectal cancers) [11, 12]. Specifically, LIF‐overexpression has been extensively documented across various types of tumors and is correlated with poor prognosis. This correlation may arise from its promotive role in regulating EMT‐associated metastasis and increasing cancer stem cell populations [12, 13]. The three transcripts of the LIF gene differ in their expression ratio and function in different cell types. Secretory LIFs (LIF‐D and LIF‐M) act mainly by secretion to promote cell differentiation and embryonic development; intracellular LIFs (LIF‐M and LIF‐T) act within the cell to promote cell proliferation and regulate the cell cycle and are involved in intracellular signaling and the maintenance of cellular homeostasis. In nasopharyngeal carcinoma, increased cytoplasmic LIF enhances metastasis via modulation of the LIFR/YAP signaling pathway and produces poorer outcomes [10, 13]. In addition, LIF impairs anti‐PD1 therapy by modulating CXCL9 expression in tumor‐associated macrophages (TAM), ultimately disrupting the infiltration of CD8^+^ T cells into the tumor microenvironment [14]. The enhanced LIF secretion has been reported to be involved in inducing cancer cell stemness via circFARP1, thus promoting gemcitabine resistance in pancreatic cancer [15]. Despite the growing number of studies on the oncogenic function of LIF in different tumors, the carcinogenic role of LIF in HCC remains unclear.

LIF has been shown to be mainly involved in the STAT pathways in HCC, promoting the JAK1/STAT3 signaling pathway to mitigate the cytotoxic effects of 5‐fluorouracil/cisplatin [16]. Previous studies have also found that decreased LIF expression, driven by miR‐637 overexpression, inhibits STAT3 phosphorylation and cancer cell growth in HCC [17]. However, limited evidence suggests that LIF activates STAT3 signaling, but studies have focused on the effects of secreted LIF and LIFR, while there is a lack of research on other effects of LIF on HCC, and small molecule inhibitors of LIF have not been established. Despite the reported small molecule inhibitors, they work by blocking the binding of LIF to LIFR, such as EC359 (PMID: 31142661) reported as a small molecule antagonist of LIF signaling, and mifepristone (PMID: 36359879). In addition, MSC‐1/AZD0171 neutralizing LIF mAb, although in Phase 2 combined human clinical trial (NCT049999690), is primarily intended for the treatment of metastatic pancreatic cancer, and there is still a lack of effective small molecule inhibitors for cytoplasmic LIF in HCC. Furthermore, the tolerance of cytokine activity to neutralizing antibodies and the identification of different transcripts of LIF also explain the presence of intracellular LIF proteins in HCC [9, 18, 19]. Therefore, further investigation of cytoplasmic LIF in HCC progression and the development of effective small‐molecule drugs targeting cytoplasmic LIF in cancer therapy represents an effective treatment strategy and clinical direction.

Cyclovirobuxine D (CVB‐D), widely used in the treatment of cardiovascular diseases, is a steroidal alkaloid derived from *Buxus microphylla*. In recent years, there has been increasing evidence that CVB‐D may exert anti‐tumor effects in a variety of cancers. CVB‐D inhibits low‐grade glioma and glioblastoma cell proliferation by inducing apoptosis and mitochondrial damage [20, 21]. Moreover, CVB‐D dampened colorectal cancer by inducing cell cycle arrest and apoptosis via the CTHRC1‐AKT/ERK‐Snail signaling pathway [22]. In HCC, the specific target of CVB‐D has not been identified, and only the inhibitory effect of CVB‐D on proliferation and metastasis has been reported, which may be related to the negative regulation of the EGFR‐FAK‐AKT/ERK1/2‐Slug signaling pathway [23]. However, its mechanism remains to be investigated and specific targets to be identified. Thus, exploring the possible utility of CVB‐D in HCC and elucidating its intricate mechanisms may provide a new direction for HCC therapy.

Here, we identified LIF as a potential biomarker and druggable target in HCC, as it inhibits HCC through the LIF/p38MAPK/p62 pathway, an unreported pathway in HCC tumorigenesis. More importantly, CVB‐D was identified as the first established cytoplasmic LIF inhibitor by biotin‐modified CVB‐D‐Probe utilizing mass spectrometry and CVB‐D was further validated to bind LIF at Valine 145. Moreover, we showed that CVB‐D suppressed HCC progression and metastasis by inducing mitophagy dependent on the LIF/p38MAPK axis both in vitro and in vivo, accompanied by cell cycle arrest. As mentioned above, we demonstrate the oncogenic role of LIF in HCC by exploiting LIF inhibition as a potential therapeutic strategy and identifying CVB‐D as the first established LIF inhibitor to improve potential HCC therapeutics.

## 2 Results

### 2.1 LIF is Identified as a Potential Druggable Target in HCC

**FIGURE 1:**
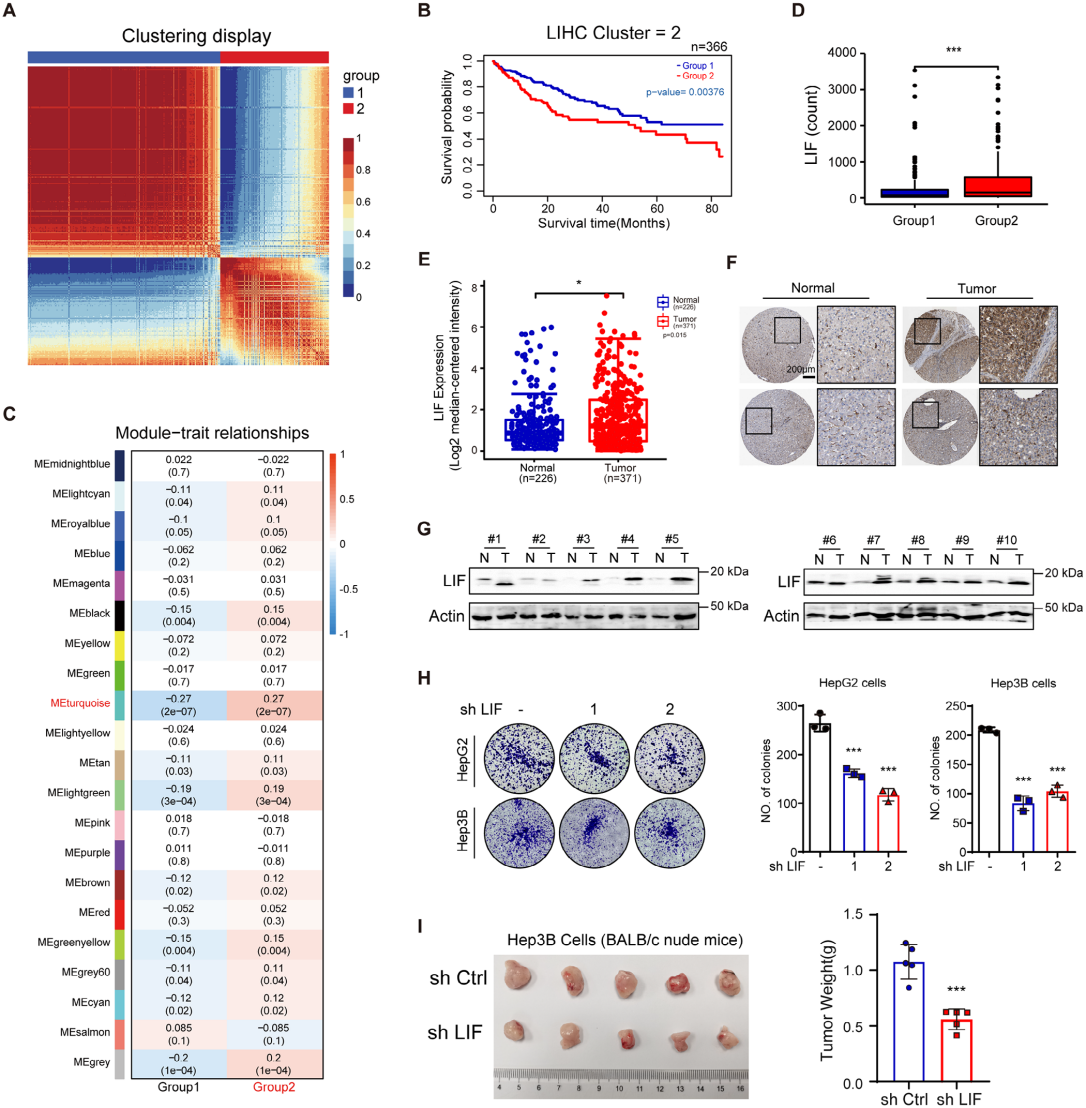
LIF as a candidate biomarker in LIHC. (A) Heatmap of patients’ similarity matrix clustered by the combined method of SNF and consensus clustering. (B) Survival curves of OS for the two subgroups clustered by the combined method of SNF and consensus clustering. (C) Heatmap of module eigengenes and traits correlation matrices with correlation and *p* value. (D) Boxplot of LIF expression in Group1 and Group2. (E) Comparison of the LIF mRNA levels between tumor and normal tissues obtained from TCGA HCC dataset. (F) Representative images of LIF expression with different levels in HCC tumor tissues and paired non‐tumor tissues. Scale bars, 200 µm. (G) Representative images of LIF expression obtained by western blotting analysis in HCC tumor tissues compared to matched NAT. T: HCC tumor tissue; N: paired NAT. Loading control was assessed by Actin. (H) Representative images of foci formed by shLIF and empty vector transduced cells in monolayer culture. (I) Knockdown of LIF in Hep3B cells reduces the subcutaneous tumors growth in Balb/c nude mice (*n* = 5 per group). *p < 0.05; ***p < 0.001. Data represent mean ± SEM.

We integrated the mRNA and miRNA expression data from patients of the TCGA‐LIHC (The Cancer Genome Atlas‐Liver Hepatocellular Carcinoma) cohort to identify potential new biomarkers or druggable targets in LIHC [24]. The consensus clustering results showed that *k* = 2–4 was the optimal number of clusters for patient classification (Figure S1A). Subsequently, to obtain more robust subtypes, we integrated the methods of consensus clustering and similarity network fusion to separate the patients into two subgroups, Group 1 (*n* = 234) and Group 2 (*n* = 132). The heat map of the similarity matrix shows the significant differences in patient characteristics between the two subgroups (Figure 1A). Overall survival (OS) analysis showed that patients in Group 2 were significantly associated with a poorer prognosis compared to Group 1 (*p* = 0.00376; Figure 1B).

To further identify the critical mRNAs in Group 2, we performed the weighted correlation network analysis (WGCNA) to analyze the LIHC mRNA expression matrix. The heat map of the module‐trait relationships showed that the genes in the turquoise module had a strong correlation with the poor prognosis of Group 2 (cor = 0.27; *p* = 2 × 10^−7^; Figure 1C and Figure S1B,C). Therefore, we speculate that LIF may be related to the occurrence and development of LIHC. We further found that the LIF expression was significantly higher in Group 2 patients than in Group 1 patients (*p* < 0.001; Figure 1D).

Simultaneously, we analyzed the transcription data available from the Gene Expression Comprehensive (GEO) database (http://www.ncbi.nlm.nih.gov/geo/) and discovered that LIF mRNA was highly expressed in HCC tissues (Figure S1D). Moreover, analysis of the TCGA and the Oncomine databases revealed that LIF expression was upregulated in HCC tissues than in the NATs (Figure 1E). LIF was also confirmed by immunohistochemistry (IHC) staining of HCC patients (https://www.proteinatlas.org/) (Figure 1F) and western‐blotting analysis of 10‐paired samples from HCC patients (Figure 1G). Compared to different HCC cell lines, both LIF mRNA and protein levels were relatively high in HepG2 and Hep3B cells (Figure S1E).

Next, given that LIF is highly expressed in HCC, we knocked down cytoplasmic LIF in HepG2 and Hep3B cell lines with either short hairpin RNA (shRNA) or siRNA to investigate the specific role of cytoplasmic LIF in tumorigenesis. The clone formation experimental results revealed that LIF silencing inhibited the proliferation of HepG2 and Hep3B cells (Figure 1H). In addition, tumor growth was significantly reduced in Hep3B‐shLIF xenograft models compared to Hep3B‐shCtrl models (Figure 1I and Figure S1F). Based on the above results, we speculated that cytoplasmic LIF may be related to the occurrence and development of LIHC. Overexpression of LIF could lead to a poor prognosis and might facilitate the progression of HCC, indicating that LIF could be a potential biomarker and drug target in LIHC.

### 2.2 Inhibiting LIF Suppresses Metastasis by Activating p38MAPK/p62‐Modulated Mitophagy

**FIGURE 2:**
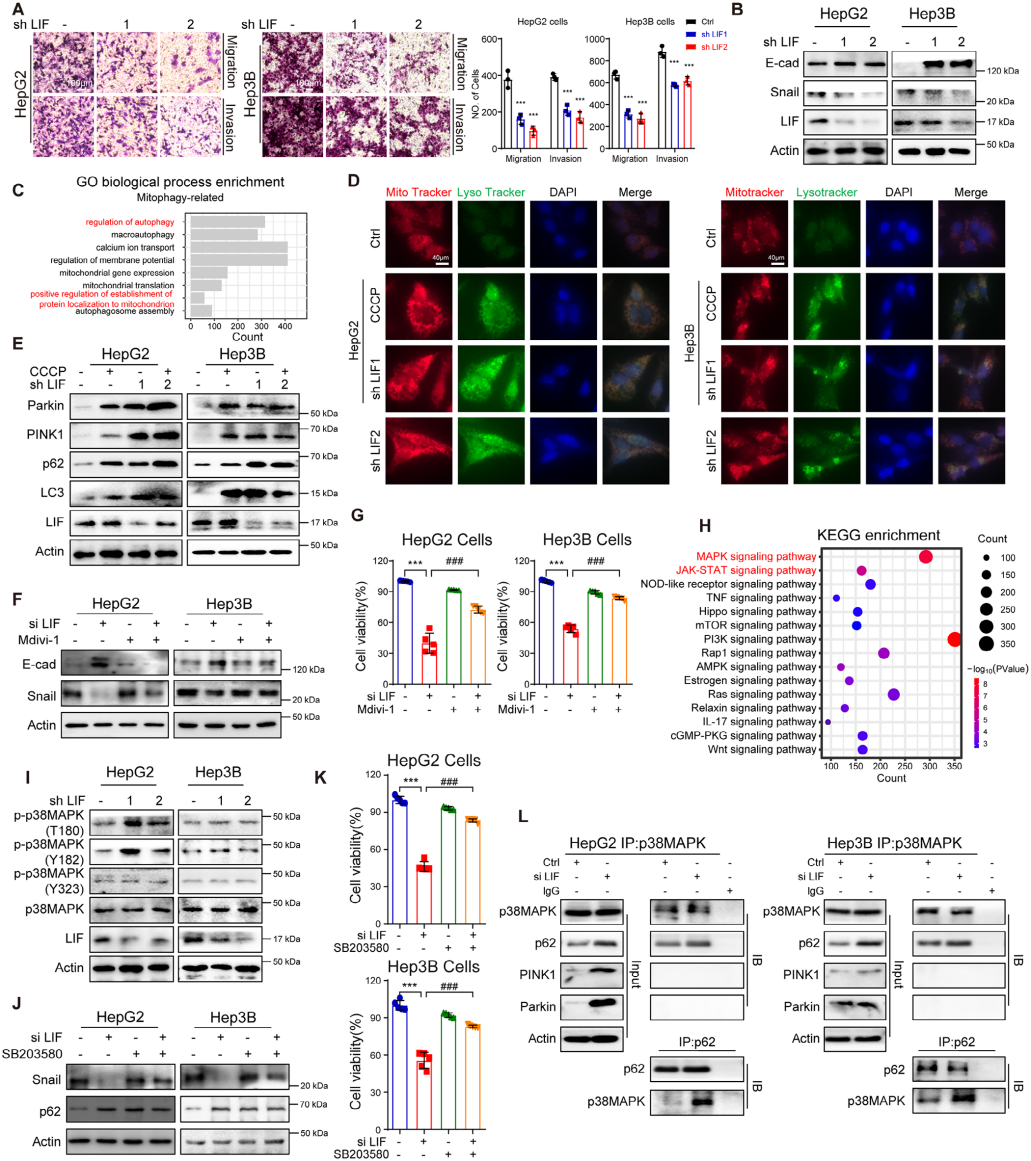
LIF plays an oncogenic role in HCC. (A) Migration and invasion capacity of cells determined in the indicated HCC cells lines. Scale bar, 100 µm. (B) Expression levels of E‐cad and Snail in cell lines with LIF knockdown. (C) Bar plot of GO biological processes enriched by the differentially expressed genes in LIHC tumor and NAT. (D) Representative images of MitoTracker and LysoTracker in HCC cells analyzed by fluorescence microscopy. Scale bar, 40 µm. (E) Expression levels of Parkin, PINK1, p62 and LC3 by western blotting in HepG2 and Hep3B cells. (F) Results of western blotting to assess expression of Snail and E‐cad with Mdivi‐1 (10 nM) treatment. (G) The results of the cell viability assay of LIF silencing when added Mdivi‐1 in HCC cell lines. (H) Bubble plot of pathways enriched by the differentially‐expressed genes in LIHC tumor and NAT. (I) The protein expression of p‐p38MAPK in cells with LIF knockdown. (J) Expression levels of Snail and p62 by western blotting in HepG2 and Hep3B cells with SB203580 treatment. (K) Cell viability of HCC cells after LIF silencing by addition of SB203580 (10 nM). (L) Co‐IP analysis of p38MAPK, p62, PINK1 and Parkin in HepG2 and Hep3B cells. The cell lysate was subjected to indicated antibodies. And the interaction between p62 and p38MAPK proteins in HepG2 and Hep3B cells. ***p <0 .001; *^###^p* < 0.001. Data represent mean ± SEM.

To further validate the oncogenic role of LIF in HCC, we first performed GO biological process analysis of differentially expressed genes in LIHC tumors and NATs. Notably, genes with biological functions related to metastasis, proliferation, cell cycle, and immunity were significantly enriched in the tumor cells (Figure S2A). Subsequently, wound‐healing and transwell assays, whether utilizing Matrigel‐coated surfaces or not, indicated that the invasive and migratory capacities of HCC cells were likewise diminished within the cytoplasmic LIF silencing (Figure 2A and Figure S2B), accompanied by alterations in EMT proteins, including decreases in Snail, and an increase in E‐cadherin (E‐cad) (Figure 2B and Figure S2C). Notably, the genes that were altered after LIF silencing focused on mitochondrial function and the autophagy process (Figure 2C). Thus, we confirmed the alterations in mitophagy and autophagy after LIF silencing, MitoTracker, and LysoTracker probes showed that LIF silencing promoted mitophagolysosome formation (Figure 2D), which were added to CCCP, a potent uncoupler, as a mitophagy‐positive control. CCCP disrupts mitochondrial membrane potential (MMP) and will trigger cellular mechanisms to recognize and sequester damaged mitochondria for degradation. The augment of LC3 suggested that activation of autophagy, while triggered by the accumulation of autophagy substrates, p62 is redirected to autophagy receptors when the increased levels of Parkin and PINK1 which Parkin recruitment of SLRs including p62. This process leads to the assembly of core autophagy machinery and the subsequent degradation of mitochondria through mitophagy [25, 26], as well as confirms the engagement of mitophagy after LIF knockdown (Figure 2E and Figure S2C) along with the dynamics of p62 (Figure S2D). Next, we added Mdivi‐1, a mitophagy inhibitor [27], to LIF knockdown cells and surprisingly observed that the decrease in Snail and the increase in E‐cad was reversed, suggesting that mitophagy plays a pivotal role in the tumorigenesis promoted by LIF (Figure 2F,G).

To address the possible intricate mechanisms of LIF, we subsequently conducted Kyoto Encyclopedia of Genes and Genomes (KEGG) pathway enrichment and found the MAPK signaling pathway to be enriched (Figure 2H). Previously, p38MAPK signaling has been reported to participate in the regulation of HCC growth [28], cell cycle progression [29], and metastasis [30]; however, the relationship between the p38MAPK signaling pathway and mitophagy has rarely been reported. Accordingly, we first investigated the regulation of LIF on p38MAPK in vitro and in vivo by knocking down LIF with either siRNA or shRNA, which resulted in an increase in phosphorylated‐p38MAPK at the Thr180/ Tyr182 sites but did not affect the level of the Tyr323 site (Figure 2I and Figure S2E). It is suggested the partial activation of p38MAPK is triggered by LIF knockdown since the phosphorylation of p38MAPK at both Thr180 and Tyr182 was 10–20‐fold more active than p38MAPK phosphorylated at Thr180 only [31]. The Thr180/ Tyr182 phosphorylated p38MAPK could sufficiently inhibit the proliferation and metastasis of HCC cells, with concomitant induction of mitophagy (Figure S2F–K). Following this, we added SB203580, a specific inhibitor of p38MAPK [32], to HepG2 and Hep3B cells after LIF knockdown and found that treatment with SB203580 reversed the effect of the EMT protein Snail (Figure 2J), the autophagic proteins p62 (Figure 2J), mitophagy (Figure S2L), and LIF knockdown on cell viability (Figure 2K). These results demonstrated the involvement of p38MAPK in the oncogenic role of LIF. To demonstrate the possible interaction of p38MAPK in modulating mitophagy, co‐IP was performed to determine whether p62, but not PINK1 and Parkin, is essential for p38MAPK induction in mitophagy (Figure 2L and Figure S2M). Altogether, these results suggest that LIF inhibition suppresses tumor growth and metastasis by activating p38MAPK/p62‐modulated mitophagy in HCC therapy.

### 2.3 CVB‐D Is Repurposed as the First Established Small‐Molecule Inhibitor of LIF

**FIGURE 3:**
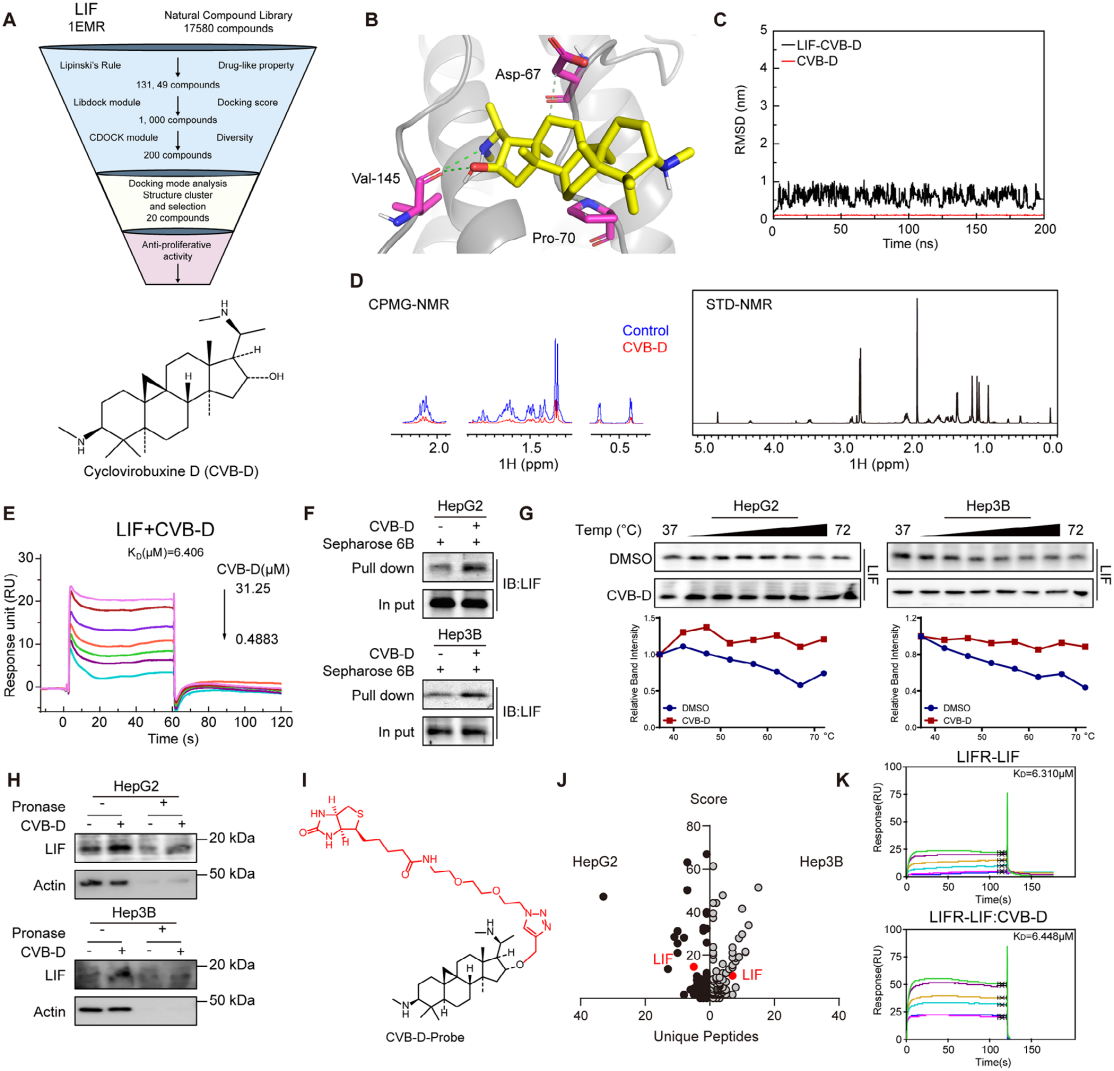
Identification of a small‐molecule targeting LIF. (A) Schematic diagram of the screening of LIF small molecule inhibitors from the natural small molecule library. And chemical structure of CVB‐D. (B) The predicted binding mode of CVB‐D and LIF. (C) Molecular dynamics simulation of CVB‐D binding to wild type LIF. The binding conformation was stabilized after a 5 ns simulation, except in the CVB‐D/LIF complex. (D) Direct interaction between CVB‐D and LIF was confirmed by STD‐NMR. (E) Direct interaction between CVB‐D and LIF confirmed by SPR analysis. (F) Verification of the interaction between CVB‐D and LIF through pull‐down assay. (G) HepG2 and Hep3B cells were exposed to 60 µM of CVB‐D, followed by cellular thermal shift assays. The targeting of LIF by CVB‐D in the cells was detected by western blotting assays. (H) CVB‐D promoted resistance of LIF to proteases (DARTs). (I) The structure of biotin‐labeled CVB‐D probe. (J) Matching score and unique peptide of identified interacting protein using chemical probe. (K) Direct interaction between CVB‐D and LIF/LIFR was confirmed by SPR analysis.

Given that inhibiting LIF leads to the regression of HCC, we initially devised a screening methodology aimed at uncovering potential compounds that selectively interact with LIF (Figure 3A and Figure S3A). In total, 17,580 compounds from the natural small‐molecule alkaloid library were established before sifting according to Lipinski's rule of five, yielding 13,149 compounds. Subsequently, we rapidly docked the crystal structure of LIF (PDB code: 1EMR) utilizing the LibDock module and obtained 1,000 hit compounds (score above 120). Next, the CDOCKER protocol was used for accurate docking of 1,000 hits, and 200 compounds were filtered out. Docking mode analysis and structure clustering were performed, and 20 candidates were selected for the anti‐proliferative activity assay. Among them, Cyclovibuxine D (CVB‐D, PubChem CID: 260439) (Figure 3A), a steroidal alkaloid derived from *B. microphylla*, was selected for its better anti‐proliferative effect than any other compound on HepG2 and Hep3B cells (Figure S3B). CVB‐D was found to bind LIF at Val145 via a hydrogen bond, and at Asp67 and Pro70 via hydrophobic interactions (Figure 3B). Stabilization of the CVB‐D/LIF complex conformation after 10 ns of simulation, small fluctuations in the root‐mean‐square deviation (RMSD) of the system, and a convergence of energy, temperature, and pressure indicate that it is a well‐behaved system (Figure 3C).

After that, purifying the un‐glycosylated recombinant LIF protein was subjected to nuclear magnetic resonance (NMR) analysis (Figure 3D) and surface plasmon resonance (SPR) analysis (Figure 3E); it showed a dissociation constant (KD) of LIF with CVB‐D was 6.406 µM, which confirmed the direct interaction of LIF with CVB‐D, and simultaneously a positive saturation transfer difference (STD) signal appeared in the STD spectra. Furthermore, we investigated the binding properties of CVB‐D and LIF. The pull‐down assay confirmed that LIF could directly interact with CVB‐D (Figure 3F), while the cellular thermal shift assay (Figure 3G) along with drug affinity responsive target stability assay demonstrated that CVB‐D directly interacted with LIF, as reflected by the significantly increased thermal stability of LIF and attenuated degradation of LIF induced by pronase in cells (Figure 3H). More importantly, CVB‐D was shown to inhibit the proliferative capacity of HCC cells after overexpression of LIF (Figure S3C,D).

To further elucidate the mechanism by which CVB‐D exerts its anti‐cancer effects through targeting LIF, we employed proximity labeling, a reliable method utilized for investigating the interactions between ligands and proteins, along with chemical proteomics to identify the cellular targets of CVB‐D [33, 34]. Herein, we designed and synthesized a biotin‐conjugated probe derived from CVB‐D (Figure 3I). Subsequently, we assessed the biological activity of the probe in HepG2 and Hep3B cells (Figure S3E,F), demonstrating that the probe has similar biological functions to CVB‐D and might share similar target sites, in proliferation and inhibition of LIF protein expression. Subsequently, we attached the probes to streptavidin‐coated magnetic beads and exposed them to a cell lysate. Next, we immobilized the probes on streptavidin magnetic beads, which were then enriched and eluted after incubation with cell lysate. Importantly, LIF was consistently captured and identified as an interacting protein using protein spectrometry in both HepG2 and Hep3B cells (Figure 3J). These findings provide evidence that LIF is indeed targeted by compound CVB‐D.

Interestingly, we found that CVB‐D could affect the distribution and expression of LIF (Figure S3G) but not the expression of the LIF receptors LIFR and gp130 (Figure S3H), because different localized LIF proteins are produced in the cell, one secreted from the cell and acting via cell surface receptors and the other localized within the cell [18], which is what we are interested in. In addition, we showed that the addition of EC359, a LIFR inhibitor [35] did not impact the inhibitory effect of CVB‐D on LIF and the protein expression of LIFR and gp130 in HCC cells (Figure S3I). What's more, we found that CVB‐D did not affect the binding of LIF and the receptor LIFR by SPR analysis (Figure 3K). Collectively, these findings indicate that CVB‐D is the first established small‐molecule inhibitor of LIF and CVB‐D can directly target LIF rather than acting on LIF or its complex with the receptors LIFR and gp130.

### 2.4 CVB‐D Induces Mitophagy via the LIF/p38MAPK/p62 Axis, Accompanied by Cell Cycle Arrest and Metastasis

**FIGURE 4:**
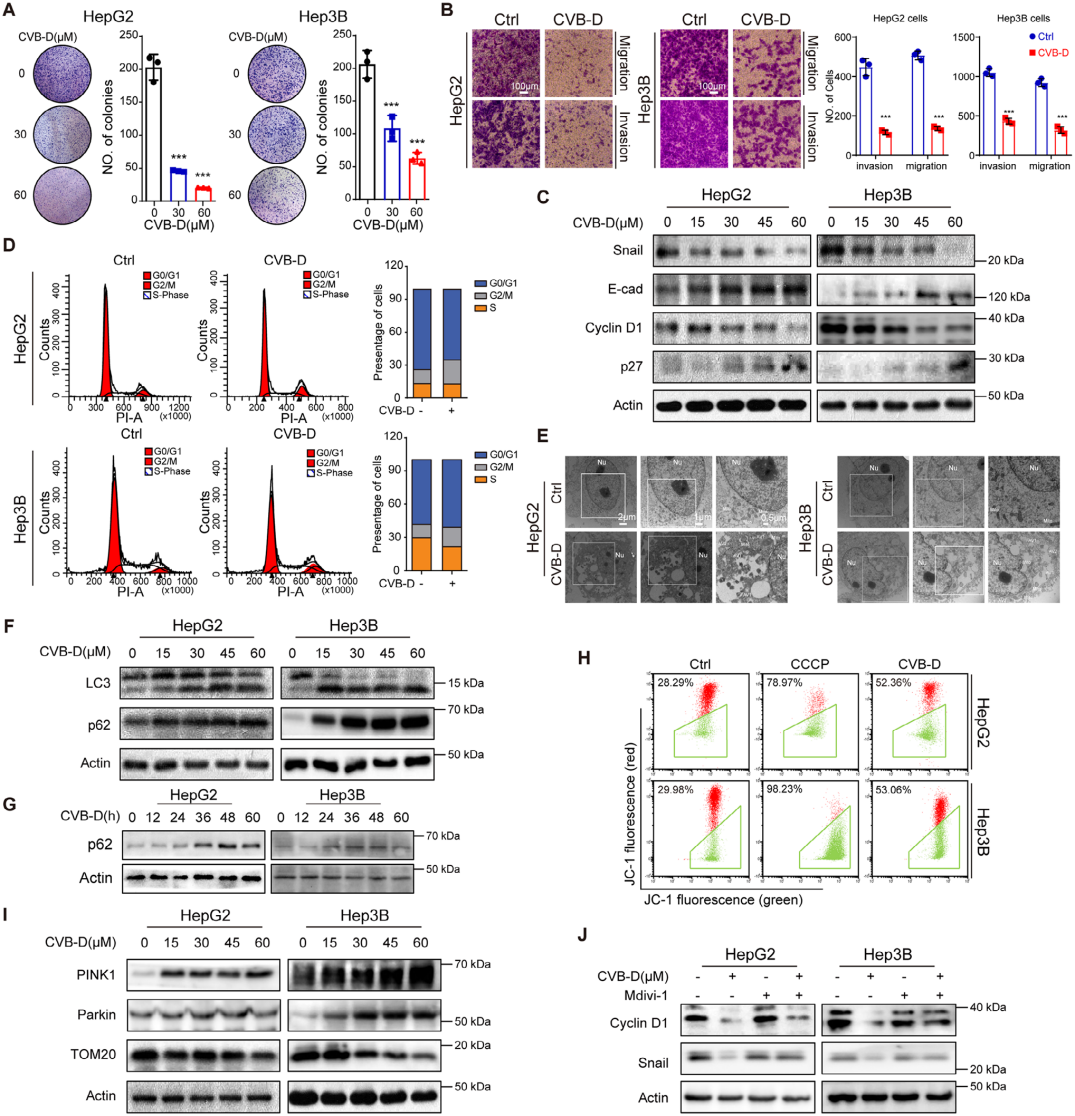
Inhibition of mitophagy attenuates CVB‐D induced cell cycle arrest and suppressed metastasis in HCC. (A) Colony formation assays showing cell proliferation of HCC cells with or without CVB‐D treatment. (B) The migration and invasion capacity of the indicated HCC cells. Scale bar, 100 µm. (C) Western blotting experiments for the expression of the indicated proteins after CVB‐D stimulation at different doses. (D) CVB‐D induces G2/M arrest, as indicated by flow cytometry. (E) TEM analysis of mitophagy in HepG2 cells and Hep3B cells. Nu: nucleus; AV1: Autophagic vacuole; AV2: Autophagic lysosomes. Scale bar, 2 µm. (F) Western blotting of LC3 and p62 in HepG2 and Hep3B cells with or without CVB‐D treatment. (G) Expression levels of p62 in cell lines with CVB‐D in time‐dependent. (H) Cell mitophagy assessed by flow cytometry in HepG2 cells and Hep3B cells with or without CVB‐D treatment. (I) Western blotting of PINK1, Parkin and TOM20 in HepG2 and Hep3B cells with or without CVB‐D treatment. (J) Changes in the metastasis and cell cycle arrest related proteins detected by western blotting after treatment of HCC cells with Mdivi‐1. ***p < 0.001. Data represent mean ± SEM.

Having established that CVB‐D functions as a LIF inhibitor with prominent anti‐tumor characteristics, we embarked on an in‐depth exploration of its efficacy and the underlying mechanisms through which it exerts inhibitory effects on HCC in vitro. Initially, we assessed the impact of CVB‐D on the proliferation of HepG2 and Hep3B cell lines and discovered that following 48 h of exposure, CVB‐D significantly reduced the viability of HCC cells (Figure 4A and Figure S4A). In addition, we observed compromised migration and invasion of HCC cells by wound‐healing and transwell assays (Figure 4B and Figure S4B), as well as corresponding alterations in EMT protein levels (Figure 4C and Figure S4C,D). Considering the involvement of LIF in modulating mitophagy in dampening HCC, we examined the effects and in‐depth mechanisms of CVB‐D on autophagy and mitophagy by inhibiting LIF in HCC, while also preliminarily impact on cell cycle arrest of CVB‐D was simultaneously detected (Figure 4C,D).

Next, we observed the elevated number of autophagosomes and vacuoles in HCC cells treated with CVB‐D indicated the activation of autophagy (Figure 4E and Figure S4E,F), which could also be proven by the dynamic increased p62 level and transition of LC3‐I to LC3‐II (Figure 4F,G and Figure S4G). Importantly, mitophagy was also induced by CVB‐D, as reflected by the decrease in MMP (Figure 4H) in addition to the upregulation of PINK1 and Parkin and the downregulation of TOM20 (Figure 4I). It was also observed in CVB‐D‐treated HCC cells, suggesting that cell cycle arrest also occurs simultaneously with the induction of mitophagy. After Midiv‐1 treatment, the induction of invasion and cell cycle arrest caused by CVB‐D was reversed, supporting the fact that CVB‐D activates mitophagy in HCC cells (Figure 4J and Figure S4H).

**FIGURE 5:**
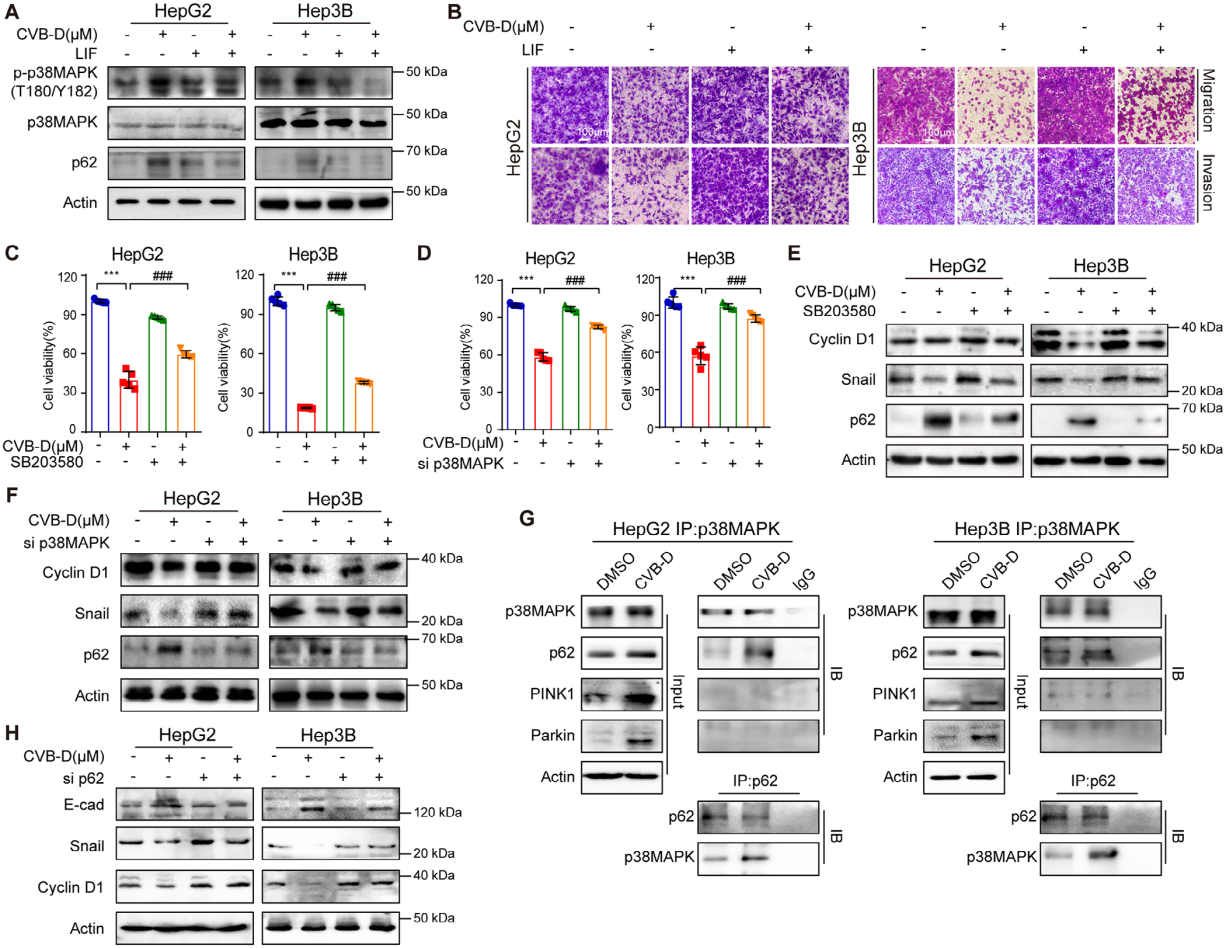
CVB‐D suppresses HCC tumorigenesis via activation of LIF/p38MAPK/p62 axis. (A) Changes in the phosphorylation levels of p38MAPK and p62 detected by western blotting of LIF‐overexpression HCC cells. (B) Transwell assays were used to investigate the cell migratory and invasive capacities of HCC cells with or without LIF‐overexpression. Scale bar, 100 µm. (C) The results of the cell viability assay of CVB‐D when added SB203580 in HepG2 and Hep3B cells. (D) MTT assays were conducted to study cell proliferation of HCC cells with or without CVB‐D for transferring siRNA of p38MAPK. (E) Expression of Cyclin D1, Snail, and p62 after addition of SB203580 in the indicated HepG2 and Hep3B cells. (F) Expression levels of Cyclin D1, Snail, and p62 that had been treated with or without CVB‐D for transferring siRNA of p38MAPK. (G) Endogenous interaction between p38MAPK and mitophagy‐related protein detected in HCC cells. IgG was used as a negative control. p62 protein interaction with p38MAPK protein was inhibited by CVB‐D. (H) Expression of E‐cad, Snail and Cyclin D1 after silencing of p62 in the indicated HepG2 and Hep3B cells. ***p <0 .001; *^###^p* < 0.001. Data represent mean ± SEM.

Phosphorylation of p38MAPK at Thr180/ Tyr182 increased after CVB‐D treatment, indicating activation of the p38MAPK pathway (Figure S5A). In addition, ectopic LIF overexpression in HCC cells reversed the CVB‐D‐induced phosphorylation of p38MAPK at the Thr180/ Tyr182 sites (Figure 5A) and reversed the anti‐tumor effect of CVB‐D (Figure 5A,B and Figure S5B), demonstrating that LIF is a potential druggable target of CVB‐D. Next, we explored the role of p38MAPK in CVB‐D‐induced mitophagy. Treatment with SB203580 and si‐p38MAPK before the addition of CVB‐D promoted anti‐tumor phenotypes (Figure 5C,D and Figure S5C–F) and reduced the expression of Cyclin D1, Snail, and p62 (Figure 5E,F). Interestingly, co‐IP showed that p38MAPK interacted with p62, but not PINK1 and Parkin, to induce mitophagy (Figure 5G and Figure S5G). Moreover, CVB‐D failed to suppress metastasis (Figure 5H) when p62 was silenced (Figure S5H), affirming the participation of p62 in the CVB‐D‐mediated LIF/p38MAPK/p62 signaling, which contributes to the suppression of HCC progression. Collectively, these results suggest that CVB‐D induces mitophagy by activating the p38MAPK/p62 pathway through targeting LIF, which may be accompanied by cell cycle arrest.

**FIGURE 6:**
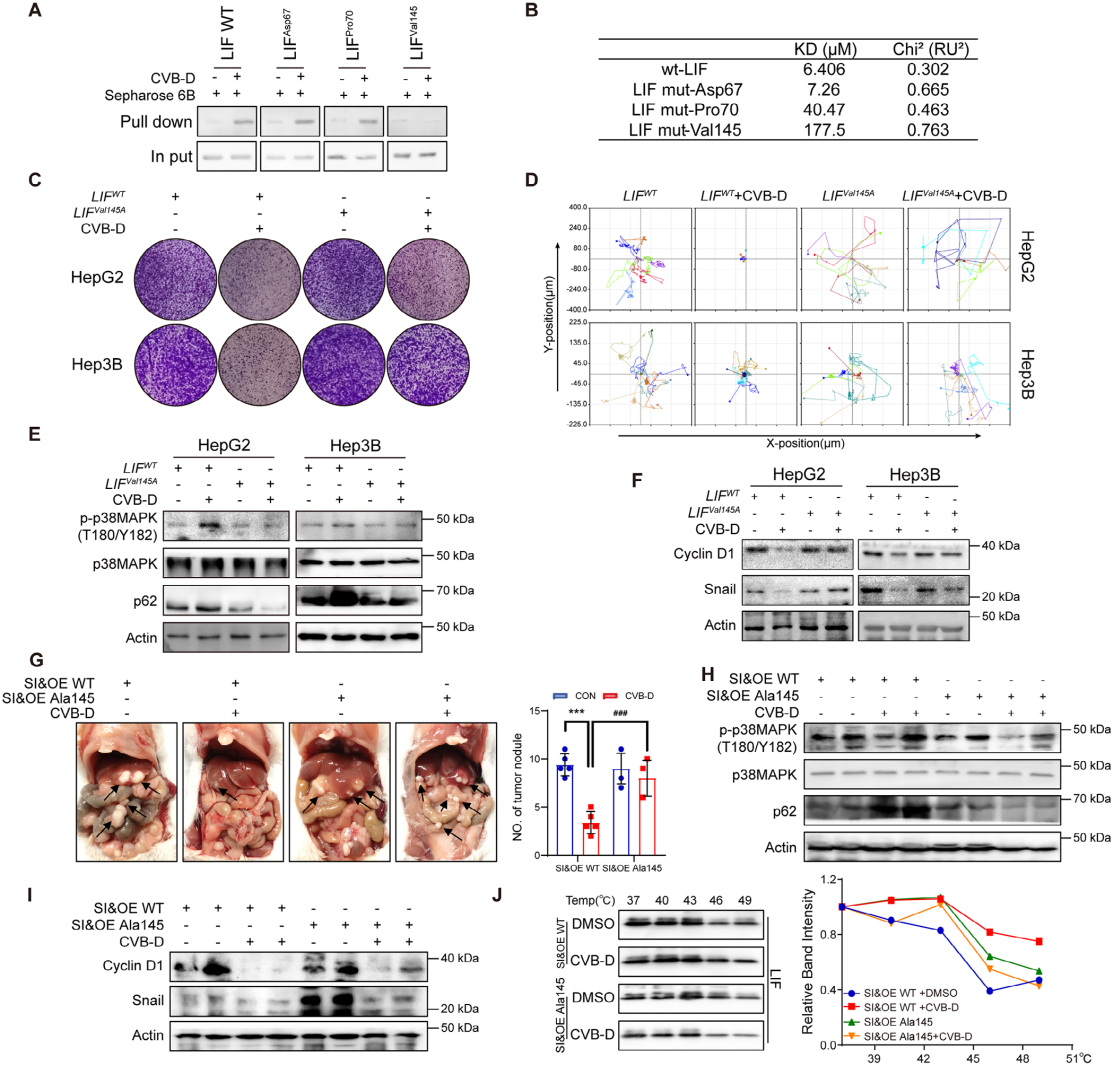
Inhibition of hepatocarcinogenesis by CVB‐D targeted LIF is facilitated by Valine 145. (A) Verification of the interaction between CVB‐D and mutant LIF through pull‐down assay. (B) SPR analysis of CVB‐D binding to LIF mutant. (C) Colony formation assays were conducted to study cell proliferation of HCC cells with or without CVB‐D in wt‐LIF and LIF‐Val145 mutant HCC cell. (D) HepG2 and Hep3B cells analyzed for single‐cell motility (6 cells per condition). (E) Western blotting to determine the phosphorylation of p38MAPK and p62 in wt‐LIF or LIF‐Val145 mutant containing HCC cells. (F) The cell lines were analyzed for the expression of Cyclin D1 and Snail by western blotting assays. (G) Measurement and comparison of the number of tumors formed in mice with targeted mutations by AAV virus to establish an orthotopic mouse model of hepatocellular carcinoma (*n* = 5 per group). (H) Changes of phosphorylation levels of p38MAPK and p62 were detected treated with CVB‐D by western blotting for wt‐LIF and LIF‐Val145 mutant of orthotopic mice. (I) The tumors tissues were analyzed for the expression of Cyclin D1 and Snail by western blotting assays (each lane represents a different tumor sample). (J) CVB‐D treatment increases the thermal stability of recombinant wt LIF in cells but not in its LIF‐Val145 mutant as measured by the temperature‐dependent cellular thermal shift assay, compared with DMSO. ***p <0 .001; *^###^p* < 0.001. Data represent mean ± SEM.

In addition, we verified the binding mode of the interactive sites between CVB‐D and LIF by designing three mutants at different positions (Asp67, Pro70, and Val145) (Figure 3B). Through pull‐down assay, we found that Val145 was critical for the binding between CVB‐D and LIF (Figure 6A). Mutations at the Val45 site effectively diminished binding between CVB‐D and LIF mutants, as confirmed by SPR analysis (Figure 6B). in vitro studies also demonstrated the vital role of Val145 in LIF as the key binding mode. CVB‐D failed to inhibit proliferation and single tumor cell motility in LIF‐Val145 site‐directed mutagenesis (Figure 6C,D and Figure S6A,B). The LIF‐Val145 mutation decreased the phosphorylation of p38MAPK at the Thr180/ Tyr182 sites, accompanied by a decline in p62 level (Figure 6E), as well as, the LIF‐Val145 mutation induced cycle block and inhibited metastasis (Figure 6F), suggesting the importance of the Val145 site of LIF for binding with CVB‐D in vitro.

Consistently, to validate the binding site of CVB‐D on LIF, we transfected orthotopic mice, either wild‐type LIF (LIF^WT^) or Val145 mutant LIF (LIF^Val145A^) via adeno‐associated virus, were used to determine the importance of the Val145 site of LIF in vivo when targeted by CVB‐D. When rescued with LIF^WT^, but not LIF^Val145A^, CVB‐D inhibited the occurrence and development of HCC in vivo (Figure 6G–I and Figure S6C–E), while significantly reducing the thermal stability of LIF^Val145A^ (Figure 6J). Altogether, these results demonstrated the key role of the Val145 site of LIF in binding with CVB‐D and then triggering mitophagy via the LIF/p38MAPK/p62 axis, accompanied by cell cycle arrest to combat HCC.

### 2.5 CVB‐D Exerts a Remarkable Therapeutic Efficacy by Targeting LIF‐Modulated Mitophagy in Orthotopic Mouse and Patient‐Derived Xenograft (PDX) Models

**FIGURE 7:**
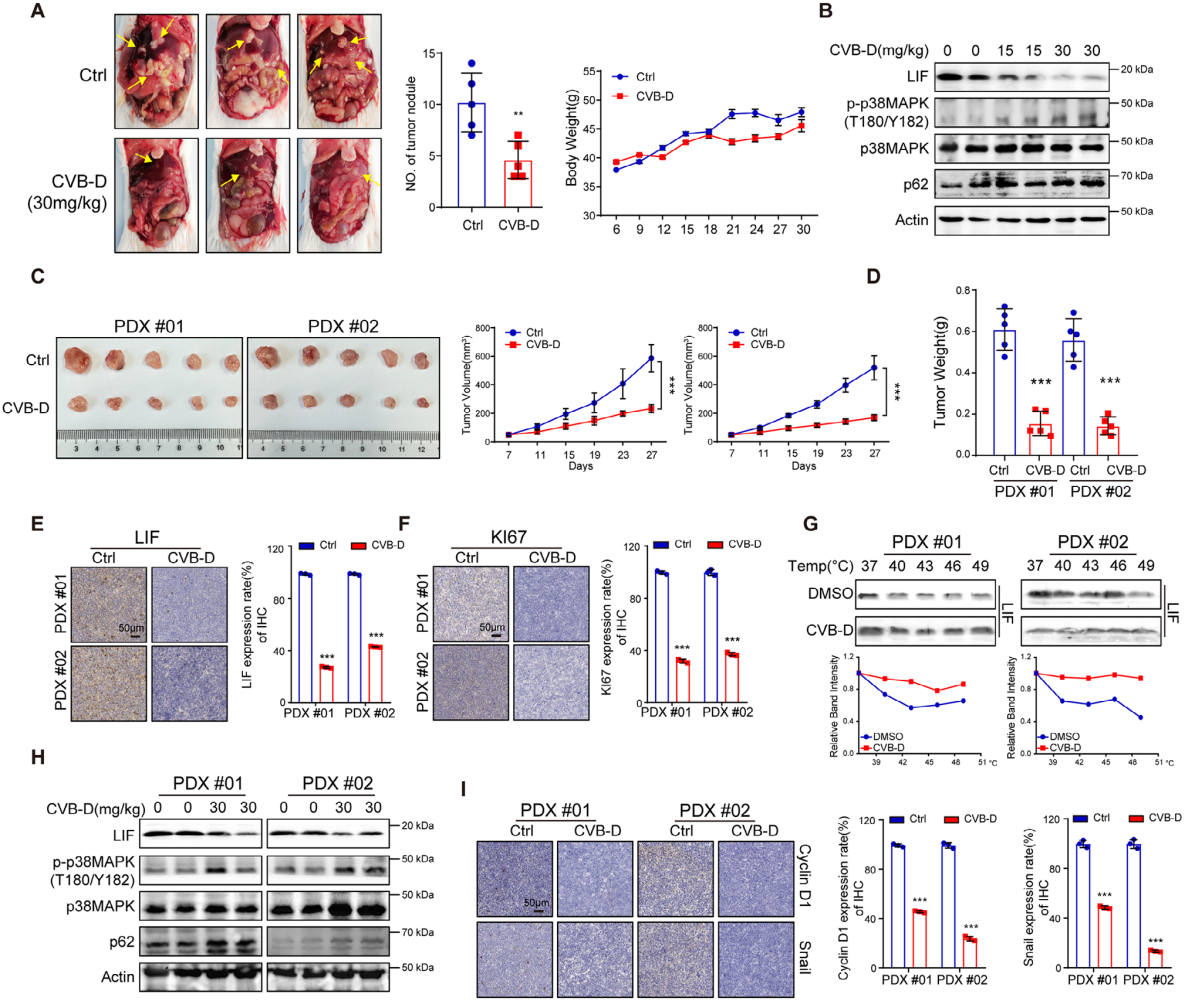
CVB‐D inhibits tumorigenesis in orthotopic mice and PDX models of HCC. (A) CVB‐D alleviated tumor growth in vivo. Representative images of orthotopic mouse tumors are shown (*n* = 5 per group). (B) CVB‐D decreases the expression of LIF and increases the phosphorylation level of p38MAPK and p62, as indicated by western blotting. (C, D) CVB‐D alleviates tumor growth in PDX. Representative images of xenograft tumors are shown (*n* = 5 per group). Tumor weight in each group is shown (*n* = 5 per group). (E) IHC staining of LIF in tumor tissues. Scale bars, 50 µm. (F) Representative IHC staining images of Ki67 in HCC tissues from the PDX model. Scale bars, 50 µm. (G) CVB‐D treatment increases the thermal stability of LIF in cell lysates as measured by the temperature‐dependent cellular thermal shift assay. (H) Changes in phosphorylation levels of p38MAPK and p62 by CVB‐D treatment detected using western blotting of HCC tissues from the PDX model. (I) Representative statistics of Cyclin D1 and Snail in HCC tissues by IHC staining. **p < 0.01;***p < 0.001. Data represent mean ± SEM.

To assess the therapeutic potential efficacy of CVB‐D in treating HCC in vivo, we initially established an orthotopic model with HCC by introducing H22 cells into KM mice through subcutaneous injection. The tumors treated with CVB‐D exhibited a substantial decrease in size when compared to the untreated control group (Figure 7A and Figure S7A). Furthermore, western‐blotting and IHC staining of tissues from the HCC orthotopic tumor group showed decreased Cyclin D1 and Snail levels and enhanced cleavage of E‐cad, p27, PINK1, and p62 in the CVB‐D group (Figure 7B and Figure S7B–D), which indicated that CVB‐D could significantly inhibit the development of HCC in vivo and is devoid of toxicity by HE staining (Figure S7E).

Moreover, we established an HCC‐PDX model to comprehensively elucidate the potential of LIF as a druggable target and its prospects for clinical translation. Initially, we detected the protein expression levels of LIF in the PDX tissues obtained from two patients and found that upregulated LIF expression was associated with high tumor malignancy (Figure 7C,D and Figure S7F,G). The results of LIF and Ki‐67 IHC staining also showed that PDX mice with high LIF expression displayed more malignant tumor proliferation than those with low LIF expression (Figure 7E,F). Consistent with the in vitro findings, CVB‐D significantly inhibited the protein expression level of LIF, while the thermal shift assay confirmed that CVB‐D could affect the stability of LIF in the two PDX mouse models (Figure 7G). Further analysis using Western blotting and IHC confirmed that CVB‐D promoted the phosphorylation of p38MAPK, suggesting its activation of the LIF/p38MAPK/p62 signaling pathway, promoted the expression of p62 protein, and promoted mitophagy in HCC (Figure 7H,I and Figure S7H,I). Importantly, the mouse body weight curve (body weight vs. time) showed no difference between the two groups in the PDX model. In addition, no evident toxicity to the organs was observed in the PDX model (Figure S7J), suggesting the preliminary safety of CVB‐D in vivo. Taken together, these results demonstrate that CVB‐D has therapeutic potential by targeting LIF‐modulated mitophagy in both orthotopic mouse and PDX models.

## 3 Discussion

LIF, a cytokine of the IL‐6 superfamily, plays intricate roles in cancer cell proliferation, migration, metastasis, and immune evasion [12, 36, 37], and has been widely reported in various types of cancer progression, including HCC [38, 39, 40]. Systematic proteomic analysis revealed that STAT3 activation and concomitant production of LIF play a promotive role in pancreatic ductal adenocarcinoma development [11]. More recently, our previous study demonstrated that the natural compound solamargine could slow HCC tumor progression by inhibiting the LIF/miR‐1/Akt signaling pathways and thereby triggering apoptosis and autophagy [39]. Moreover, we have further discovered that LIF is capable of accelerating sorafenib resistance to impede treatment, which suggests the possible oncogenic role of LIF in HCC tumorigenesis [41]. In this study, using multi‐omics approaches, we identified that LIF overexpression correlates with poor prognosis of HCC, thereby indicating that LIF is a potential biomarker of HCC.

Moreover, we demonstrated the oncogenic role of cytoplasmic LIF in activating p38MAPK/p62‐modulated mitophagy in HCC, establishing LIF as a druggable target for HCC therapy. Furthermore, MSC‐1/AZD0171 neutralizing LIF mAb promotes anti‐tumor inflammation and slows tumor growth by modulating TAM and inhibiting cancer stem cells. Although a Phase 2 combination human clinical trial is underway, primarily for the treatment of metastatic pancreatic cancer, effective small molecule inhibitors targeting LIF in HCC are still lacking. LIF has been widely reported to activate the STAT3 protein, thereby exacerbating tumorigenesis [35, 42, 43]. Specifically, in the context of lung cancer, bone marrow‐derived mesenchymal stem cells secrete LIF, which subsequently engages the LIFR/p‐ERK/pS727‐STAT3 signaling pathway, thereby modulating the balance between EMT and its reverse process, MET [44]. Notably, LIF‐induced STAT3 signaling also participates in HCC development, exemplified by arsenic trioxide, which simultaneously suppresses the LIF/JAK1/STAT3 and NF‐kB signaling pathways, thereby promoting cancer stem cell differentiation and impeding HCC development [16]. In spite of the widespread involvement of LIF in the STAT3 pathway found in oncological studies, other oncogenic pathways associated with LIF exist, for instance, the LIF/p‐AMPK signaling pathway in HCC [38], and the LIF/STAT3/ID1/ MDM2 signaling pathway in colorectal cancer [45]. In normal studies, impaired autophagy results in an accumulation of p62, making it an indicator of autophagic flux, alongside LC3B levels. Notably, p62 undergoes notable degradation during periods of active autophagic flux, while p62 levels significantly rise when the autophagic flux is obstructed or mitophagy occurs, confirming the dynamic changes of p62 in our study. Hence, the concurrent elevation of LC3B and p62 expression levels is generally regarded as a distinctive indicator of incomplete autophagy [46]. p38MAPK signaling pathway can mediate a variety of stress, inflammatory factors, and growth factor‐induced responses, and is involved in the regulation of cell proliferation, differentiation, carcinogenesis, metastasis, and apoptosis. During stress conditions, LIF assists cells in coping with stress by regulating the metabolic environment or cytoskeletal stability, while p38MAPK is activated to regulate the expression of antioxidant enzyme genes in the cell. In the present study, we found a new p38MAPK pathway enriched in KEGG analysis in response to LIF overexpression in HCC and verified that its downstream effectors, such as p62, collectively evoke mitophagy thereby facilitating malignancy.

As LIF inhibition holds promise in targeted HCC therapy, we aimed to discover an LIF inhibitor while evaluating its anti‐tumor effect and illuminating its mechanisms by targeting LIF. Previous studies have reported some natural compounds, such as stachydrine hydrochloride (SH), which exerts tumor suppressive effects in HCC by suppressing LIF expression while increasing p‐AMPK to evoke autophagy as well as cell cycle arrest [38]. While preliminary research into the anti‐tumor mechanism of SH in HCC has focused on its role in inhibiting LIF expression, it has not been definitively established that SH directly targets LIF, rendering it an LIF modulator instead of an LIF inhibitor. Here, we established CVB‐D as the first cytoplasmic LIF‐targeted inhibitor for the therapeutics of HCC, using screening and molecular docking. Furthermore, we fished for LIF with biotin‐modified CVB‐D‐Probe by mass spectrometry. As a repurposed drug candidate, CVB‐D has been found to exert anti‐tumor effects in various types of cancers, including breast cancer, gastric cancer, lung cancer, glioblastoma, and importantly, HCC [20, 40–42]. CVB‐D can trigger mitophagy via the p65/BNIP3/LC3 axis in lung cancer cells, thereby inhibiting lung cancer [49].

As for HCC therapy, CVB‐D primarily attenuated the proliferation and metastasis of HCC cells by regulating the EGFR‐FAK‐AKT/ERK1/2‐Slug signaling pathway and then inducing apoptosis and cell cycle arrest [23]. Notwithstanding, the precise target of CVB‐D and its in‐depth anti‐tumor mechanism remains elusive, which represents the main investigative challenge of repurposed drug candidates. In addition, how CVB‐D acts and its full cancer‐treatment potential await comprehensive clarification. Likewise, it gives us a hint to go further in this study, we repurposed CVB‐D as a potential drug candidate for HCC therapy, in which we not only detected LIF using mass spectrometry on biotin‐modified CVB‐D‐Probe but also found that CVB‐D may target LIF at Val145 via forming a hydrogen bond, which was demonstrated by the loss of efficacy by sh‐LIF and mutant LIF^Val145^ both in vitro and in vivo. Accordingly, we elucidated that CVB‐D induced mitophagy by triggering the LIF/p38MAPK/p62 pathway, which was mainly responsible for its anti‐tumor effect, concomitant with cell cycle arrest. CVB‐D also exhibited good efficacy and negligible toxicity in vivo, both in an orthotopic mouse model and an HCC PDX model, representing a promising candidate for further optimization and transduction in future clinical use.

## 4 Conclusion

In summary, we identified LIF as a tumor promoter in HCC and systematically investigated its oncogenic role in facilitating cancer cell growth and metastasis by modulating mitophagy through a new signaling pathway of LIF/p38MAPK/p62. More importantly, we established the first cytoplasmic LIF inhibitor, CVB‐D, which interacted with LIF at Val145 and showed promising efficacy in HCC in different mouse models, by inducing LIF/p38MAPK/p62‐dependent mitophagy. Our findings provide new insights into the role of LIF/p38MAPK/p62‐regulated mitophagy in HCC tumorigenesis and shed light on exploiting the first established LIF inhibitor CVB‐D as a small‐molecule drug candidate for future HCC therapeutics.

## 5 Materials and Methods

### 5.1 Cell Viability and Cell Cycle Distribution Analysis

HCC cells were exposed to CVB‐D (purity > 98%, Shanghai Yuanye Bio‐Technology) for 48 h, and further maintained with MTT (Sigma‐Aldrich), and absorbance was measured using a microplate reader. For colony formation assay, cells were seeded into 6‐well plates and incubated overnight, and exposed to CVB‐D for 48 h, were formed on Day 14. For the cell cycle distribution assay, cells were exposed to 60 µM CVB‐D. Cells were harvested and analyzed for cell cycle distribution by Attune NxT flow cytometry (Thermo Fisher) after staining with PI (Thermo Fisher).

### 5.2 Patients and Samples

All patients participating in our study were duly informed and provided their written consent, with the entire study undergoing rigorous approval by the Ethics Review Board of the First Affiliated Hospital, College of Medicine, Zhejiang University. A concise overview of the key characteristics of the HCC patients involved in our study is provided in Table S1.

### 5.3 Patient‐Derived Xenografts Mouse Model

Obtained fresh patient specimens (P0 = passage zero) were serially transplanted into the interscapular region of 6‐ to 8‐week‐old female NOD‐scid gamma (NSG) mice. Successful PDX transplantation was defined as stable passages to P3, followed by randomization into two groups (*n* = 8, per group) receiving saline or CVB‐D (30 mg/kg every two days) by intravenous injection for a total of 21 days. Patient information is provided in Table S2.

### 5.4 Orthotopic Mouse Model

KM mice were injected subcutaneously with H22 cells. Once tumors reached 1 cm^3^, they were excised, fragmented, and orthotopically implanted into mice livers. After two weeks, mice were divided into three groups and treated with saline or CVB‐D (15/30 mg/kg) via i.p. injection for 30 days. Mice were euthanized, and the liver was examined for tumor nodules via microscopy and histopathology.

### 5.5 SPR Analysis

Competition binding kinetics was carried out at 25°C using a Biacore T200 SPR instrument and LIF‐immobilized CM5 sensor chips as previously described [44]. Protein samples were immobilized on the chip via amine coupling chemistry in 10 mM sodium acetate at pH 4.5. Ligand solutions were injected as analytes. Data was analyzed using Biocore evaluation software (T200 Version 2.0). Interaction between CVB‐D and LIF was fitted to the steady‐state affinity model to obtain results and equilibrium dissociation constants (KD = kd/ka) were calculated.

### 5.6 STD NMR Analysis

Utilized standard NMR methodology to probe small molecule‐protein interactions as previously described. Specifically, NMR titration was acquired on a Bruker Avance NEO‐400 MHz spectrometer, which was equipped with a cryogenically cooled probe (Bruker biospin). The sample contained CVB‐D and the recombinant LIF protein dissolved in phosphate buffer for NMR data acquisition and analyzed using the Bruker TopSpin 2.1 software.

### 5.7 Immunoprecipitation and Pull‐Down Assay Analysis

The Immunoprecipitation assay and pull‐down analysis were performed as described previously [50, 51]. Samples were lysed in a buffer for immunoprecipitation. Primary antibodies were mixed with protein A/G agarose for 2 h, then incubated with cell lysates overnight. Proteins were extracted with SDS buffer, and heated at 95°C for 10 min. For pull‐down analysis, the CVB‐D was immobilized on Sepharose 6B beads (Solarbio) and incubated with lysates overnight at 4°C.

### 5.8 RNA Transfection and Viral Infection

RNA interference cells were transfected with 50 nM siRNAs using Lipofectamine 2000 reagent (Thermo Fisher) for 48 h (Table S3). For overexpressing LIF lentiviruses (GeneChem), human LIF cDNA without the 3′‐UTR region was cloned into the GV492‐GFP‐IRES retroviral vector at the specific BamHI and AgeI restriction sites. Following standard procedures, lentiviruses were generated in 293T cells and used to transduce HCC cells. For the verification mechanism experiments, cells were pre‐cultured with 10 nM SB203580 (Sigma‐Aldrich) or Mdivi‐1 (Selleck) for 1 h, then exposed to shRNA or siRNA.

### 5.9 Cell Metastasis Assays

Cells were scratched at 90%–100% confluence. Images of the wound were captured at 0‐ and 48‐h post‐scratch with CVB‐D treatments (5, 15 µM), using an Olympus camera. Assays were replicated three times. HCC cell imaging over 24 h was conducted with a Holo Monitor M4 cytometer, capturing every 10 min. Over 30 cells were manually tracked in six independent experiments. Quantitative parameters like cell speed and total distance traveled were analyzed, as previously described [52]. Cells starved overnight were seeded onto Boyden chambers with an 8 µm pore membrane. After incubation with CVB‐D (15 µM) for 48 h, adhering cells were fixed, and non‐adhering cells were removed, stained, and counted. The invasion assay followed similar steps but with an upper chamber precoated with matrigel.

### 5.10 Western Blotting Assays

Standard western blotting assays were analyzed as conducted as previously described [53]. Full scans of western blotting assays were shown in Table S4. For cellular thermal shift assays, cells pretreated with DMSO or CVB‐D (60 µM) for 24 h were lysed by heating at different temperatures for 5 min, and protein precipitates were analyzed by Western blotting.

### 5.11 Immunofluorescence and IHC Assay

In brief, human HCC cells were fixed with 4% paraformaldehyde for 30 min, followed by incubation with 0.5% Triton X‐100, and blocked with 5% BSA for 30 min. Slides were incubated with antibodies overnight at 4°C, then with secondary antibodies for 1 h. Nuclear was stained with DAPI and visualized under a fluorescent microscope (Nikon). Tissue sections were excised, formalin‐fixed, paraffin‐embedded, and then incubated with antibodies overnight at 4°C, followed by a biotin‐conjugated secondary antibody. Images were visualized by a Leica DM4000B microscope (Leica).

### 5.12 Virtual Screening

3D structures of CVB‐D were downloaded from PubChem Compound. The structure of LIF (PDB code: 1EMR) and the 3D crystal structure of LIF were obtained from the protein data bank (http://www.rcsb.org/pdb/home/home.do). We selected a screening library containing 17,580 small molecule compounds, which were energy minimized by Accelrys Discovery Studio (version 3.5) molecular modeling software utilizing the CHARMm force field. Semi‐flexible docking was performed via LibDock and CDOCKER protocols as a docking method to rearrange the top 20 small molecule compounds. Other parameters were configured on default values.

### 5.13 AAV9 Virus and Gene Delivery in vivo

AAV9 virus was produced following standard protocols. AAV9‐LIF and AAV9‐LIF‐Val145 were generated using an AAV9 vector with a liver‐specific promoter to drive the expression of LIF and LIF‐Val145 in mouse liver, respectively. AAV9‐Tbgp‐GFP (AAV‐CTRL) served as a control. The target sequences were inserted between BamHI sites downstream of the Tbg promoter in the AAV backbone. Viral genomes were quantified using dye‐based qPCR. Mice were injected with 100 µL of virus containing 10¹¹ AAV9 genomes via the tail vein. For LIF silencing, an AAV9‐U6‐LIF shRNA vector was constructed with the U6 promoter driving LIF shRNA expression. An empty plasmid was used as a negative control. LIF‐Val145 expression was maintained by injecting 100 µL of virus every four weeks [54].

### 5.14 Consensus Clustering and Similarity Network Fusion Analyses

Before clustering, we used gdc‐client to download LIHC mRNA and miRNA data from TCGA, forming the TCGA‐LIHC cohort. We selected the top 1500 variable mRNAs and top 500 variable miRNAs using MAD and normalized data via Pearson correlation distance. We then used Consensus Cluster Plus (R version 4.1, package version 1.58.0) with 80% resampling, 50 iterations, and a maximum of four clusters to determine the optimal clustering [55]. Finally, we used SNF and consensus clustering in Cancer Subtypes (version 1.20.0) to divide patients into two subgroups and analyze their survival curves. Clinical data were also downloaded from TCGA to further stratify patients according to LIF mRNA upregulation thresholds (z‐score ds (z‐score data were also dow–Meier survival curves.

### 5.15 Weighted Correlation Network Analysis (WGCNA)

To identify mRNAs associated with poor prognosis in LIHC patients, WGCNA (R package, version 1.70–3) was used to analyze the LIHC mRNA expression matrix [56], with Group 1 and Group 2 as traits. A Soft Threshold (β value) of 11 was chosen for network weighting calculation to achieve a scale‐free distribution (*R* ^2 ^> 0.9). This resulted in 21 modules, and the module most associated with poor prognosis was identified. Cytoscape software (version 3.8.2) was then used to construct the gene co‐expression regulatory network [57, 58].

### 5.16 Differential Expression and Gene Function Annotation Analysis

We used DESeq2 (version 1.32.0) to identify DEGs between LIHC tumor and NAT [59]. Under the premise that the false discovery rate (FDR) is less than 0.05, genes with log2FoldChange >1 and log2FoldChange ←1 were regarded as upregulated and downregulated genes, respectively. The lfcShrink function was used to refine estimates for low‐count or high‐dispersion genes [60]. Next, we performed GO and KEGG enrichment analyses using clusterProfiler (version 4.2.2) to explore the biological processes and pathways enriched by DEGs [61]. Results were visualized with ggplot2 (version 3.3.5) [62].

### 5.17 Statistical Analysis

Data were reported as mean ± SEM from at least three separate trials. The statistical differences in animal experiments in response to CVB‐D treatment were analyzed by one‐ or two‐way ANOVA, followed by Student's *t*‐test. All other *p* values were performed using Student's *t* test (unpaired, two‐tailed) and the statistical variation (*p* < 0.05) was considered significant.

## Author Contributions

H.Y. and Y.S. conceived and designed the experiments; Y.S. and W.J. performed the experiments; L.F., D.L., and S.C. contributed reagents and materials; Y.S., T.W., L.H., and W.J. wrote the manuscript. All authors have read and approved the final manuscript.

## Ethics Statement

We thank all the patients who donated samples for this study. All patients participating in our study were duly informed and provided their written consent, with the entire study undergoing rigorous approval by the Ethics Review Board of the First Affiliated Hospital, College of Medicine, Zhejiang University (Approval NO. 2019–402). This study was approved by the committee, and informed written consent was obtained from each patient. All procedures of animal experimental were performed under sterile conditions at Tianjin university of Traditional Chinese Medicine specified‐pathogens free facility. The principles and experimental protocols of animals used were approved by the Animal Care and Use Committee of Tianjin University of Traditional Chinese Medicine (Approval NO. TCM‐LAE2021251 and TCM‐LAEC2022012).

## Conflicts of Interest

The authors declare no conflicts of interest.

## Supporting information



Supporting Information

## Data Availability

No datasets were generated or analyzed during the current study.
