# Supplementary material for: Targeting LIF With Cyclovirobuxine D to Suppress Tumor Progression via LIF/p38MAPK/p62‐Modulated Mitophagy in Hepatocellular Carcinoma
Source: MedComm (2020). 2025 May 24;6(6):e70227. doi: 10.1002/mco2.70227 (PMC12103653; doi:10.1002/mco2.70227)
Supplement: Supplementary file 1 — Supporting Information [file MCO2-6-e70227-s001.docx]

Supplementary Materials for

**Targeting LIF with Cyclovirobuxine D to suppress tumor progression via LIF/p38MAPK/p62-modulated mitophagy**

**in hepatocellular carcinoma**

Yingying Shao *et al.*

*Corresponding author. Email: leilei_fu@163.com (L.L.F.); hyyu@tjutcm.edu.cn (Y.H.Y.).

**This PDF file includes:**

Figs. S1 to S7

Tables S1 to S3

Figure S1.


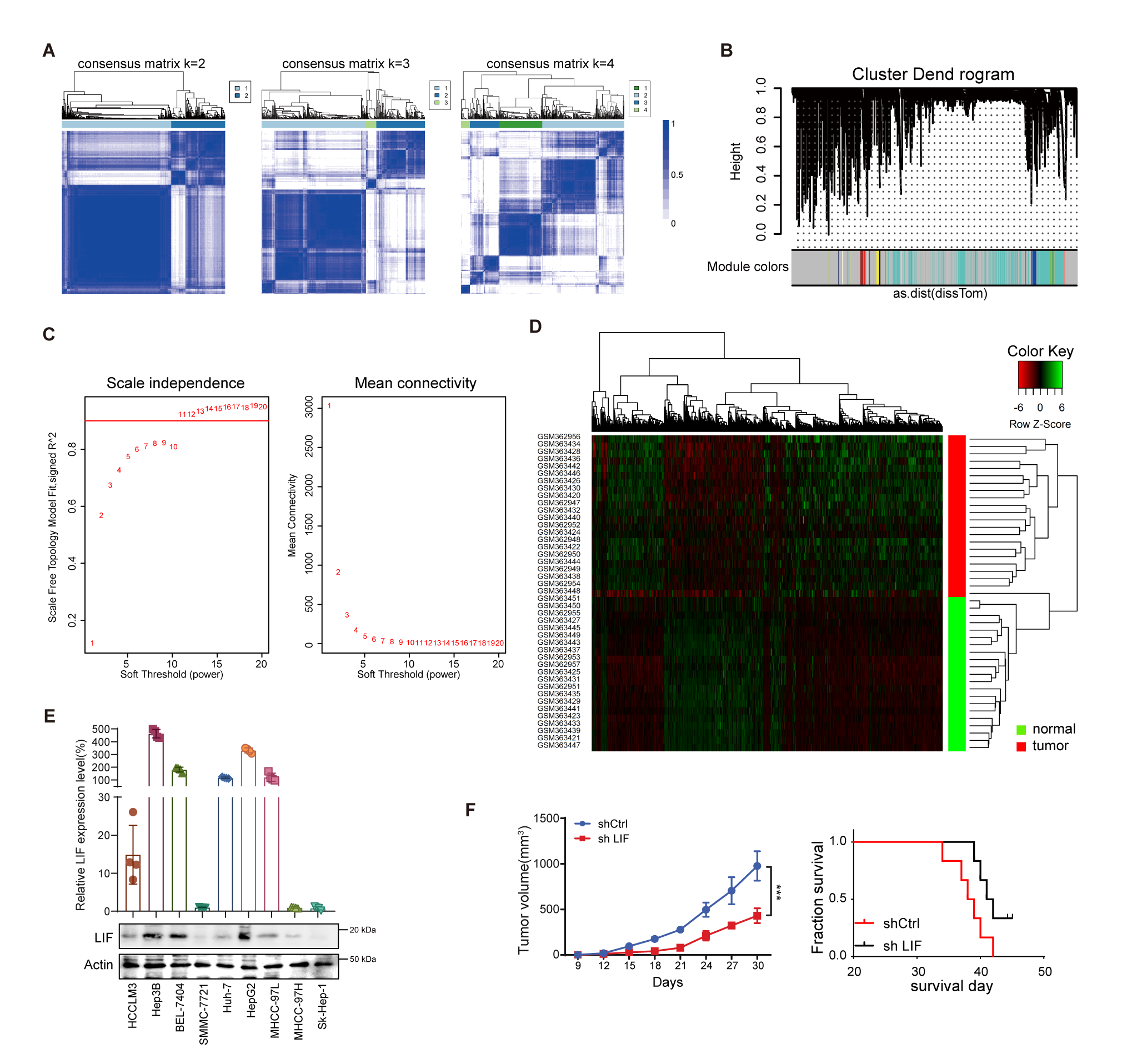


**Figure S1. LIF is overexpressed in HCC and serves as a prognostic predictor** (related to **Fig.1**)**.** (**A**) Consensus clustering subtype matrices when k =2-4. (**B**) Clustering dendrogram of genes in modules of each color. (**C**) Analysis of evaluation parameters of scale-free networks and mean connectivity for various soft-thresholding powers. (**D**) Heat map of the global mRNA expression profile in non-tumor tissues (n = 22) and hepatocellular carcinoma (HCC) tissues (n = 21) in GSE14520 dataset. (**E**) Relative protein expression of LIF in HCC tumor cell lines obtained by western blotting analysis and PCR (n = 4 per group). (**F**) Knockdown of LIF in Hep3B cells reduces the subcutaneous tumors growth in Balb/c nude mice. ****p* < 0.001. Data represent mean ± SEM.

Figure S2.


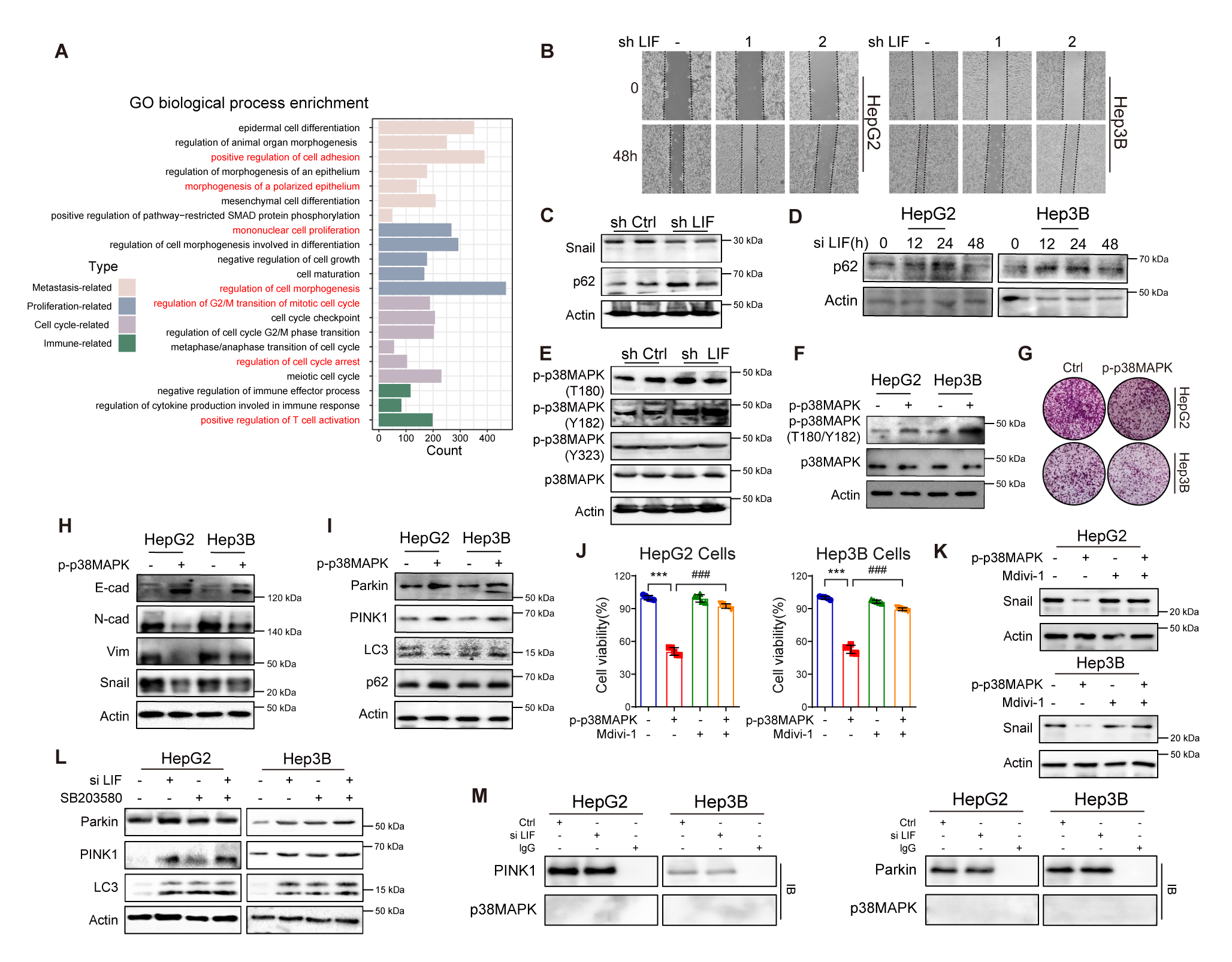


**Figure S2. LIF silencing and Overexpression p-p38MAPK(T180/Y182) weaken HCC cells’ tumorigenic ability** (related to **Fig.2**)**.** (**A**) Bar plot of GO biological processes enriched by the differentially expressed genes in LIHC tumor and NAT. (**B**) Wound healing assays showed the migration capacity of indicated HCC cells. (**C**) Expression levels of Snail and p62 in tumors tissues with LIF knockdown. (**D**) Expression levels of p62 in cell lines with LIF knockdown in time-dependent. (**E**) Knockdown of LIF was verified by western blotting assay to activate the phosphorylation activity of p38MAPK at the Thr180/ Tyr182 sites, but not at the Tyr323 site in vivo. (**F**) Expression of p-p38MAPK overexpression verified by western blotting. (**G**) Representative images of foci formed by p-p38MAPK overexpression transduced and empty vector transduced cells in monolayer culture. (**H**, **I**) Expression levels of E-cad. N-cad, Vim, Snail, Parkin, PINK1, LC3 and p62 in cell lines that had been treated with p-p38MAPK-overexpression. (**J**) The results of the cell viability assay of p-p38MAPK-overexpression when added Mdivi-1 in HCC cell lines. (**K)** Expression levels of Snail by western blotting in HepG2 and Hep3B cells. **(L**) Changes in the mitophagy related proteins were detected by western blotting for treating with SB203580 in HCC cells. (**M**) PINK1 and Parkin protein interaction with p38MAPK protein was verified by western blotting. ****p* < 0.001; *^###^p* < 0.001. Data represent mean ± SEM.

Figure S3.


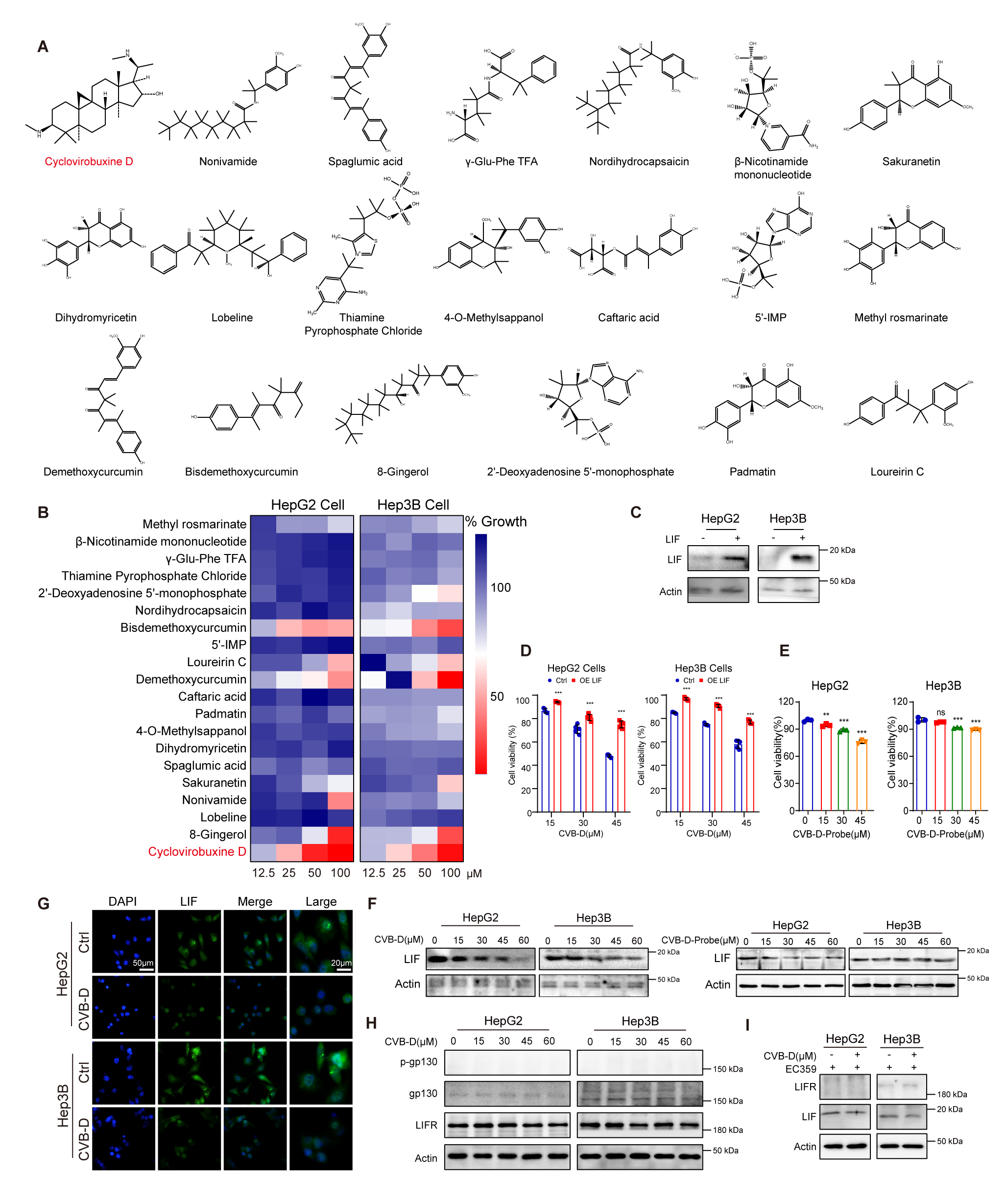


**Figure S3. Identification of a small-molecule targeting LIF** (related to **Fig.3**)**.** (**A**) Screening of LIF small molecule inhibitors from the natural small molecule library. (**B**) Heatmap of screen drug survival in two cell lines. (**C**) Western blotting verified the overexpression effect of LIF. (**D, E**) The results of the cell viability assay of CVB-D or CVB-D-Probe in HCC cell lines. (**F**) Expression levels of LIF in cell lines that had been treated with CVB-D and CVB-D-Probe. (**G**) Representative statistics of LIF by IF staining. DAPI was used for nuclear staining. Scale bar, 50 μm. (**H**) Expression levels of p-gp130, gp130, and LIFR in cell lines that had been treated with CVB-D. (**I**) Expression levels of LIFR and LIF in cell lines that had been treated with EC359. ns, no significant. ***p* < 0.01; ****p* < 0.001. Data represent mean ± SEM.

Figure S4.


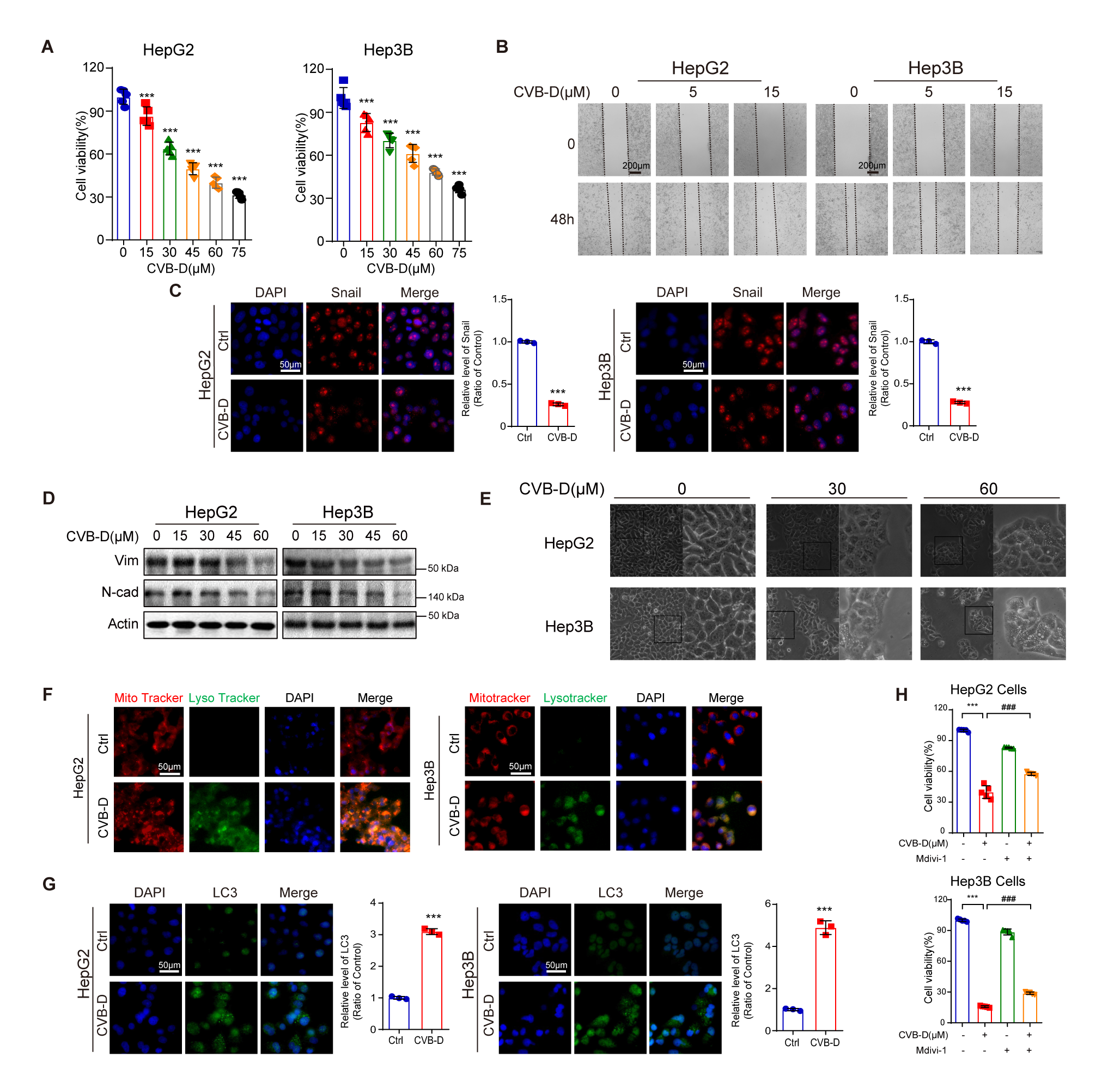


**Figure S4. CVB-D inhibition HCC metastasis and induction cell cycle arrest via mitophagy in vivo and in vitro** (related to **Fig.4**)**.** (**A**) The results of the cell viability assay of CVB-D in HCC cell lines. (**B**) Wound healing assays showed the migration capacity of indicated HCC cells. Scale bar, 200 μm. (**C**) Representative image of Snail (red) in HepG2 and Hep3B cells by IF staining. Scale bar, 50 μm. (**D**) Expression levels of EMT-related protein in HepG2 and Hep3B cells with or without CVB-D. (**E)** Observe cell morphology changes under a microscope with or without CVB-D. **(F**) Representative images of MitoTracker and LysoTracker in HCC cells analyzed by fluorescence microscopy. Scale bar, 50 μm. **(G**) Representative image of LC3 (green) in HepG2 and Hep3B cells by Immunofluorescence staining. Scale bar, 50 μm. (**H**) The results of the cell viability assay of CVB-D when added Mdivi-1 in HCC cell lines. ****p* < 0.001; *^###^p* < 0.001. Data represent mean ± SEM.


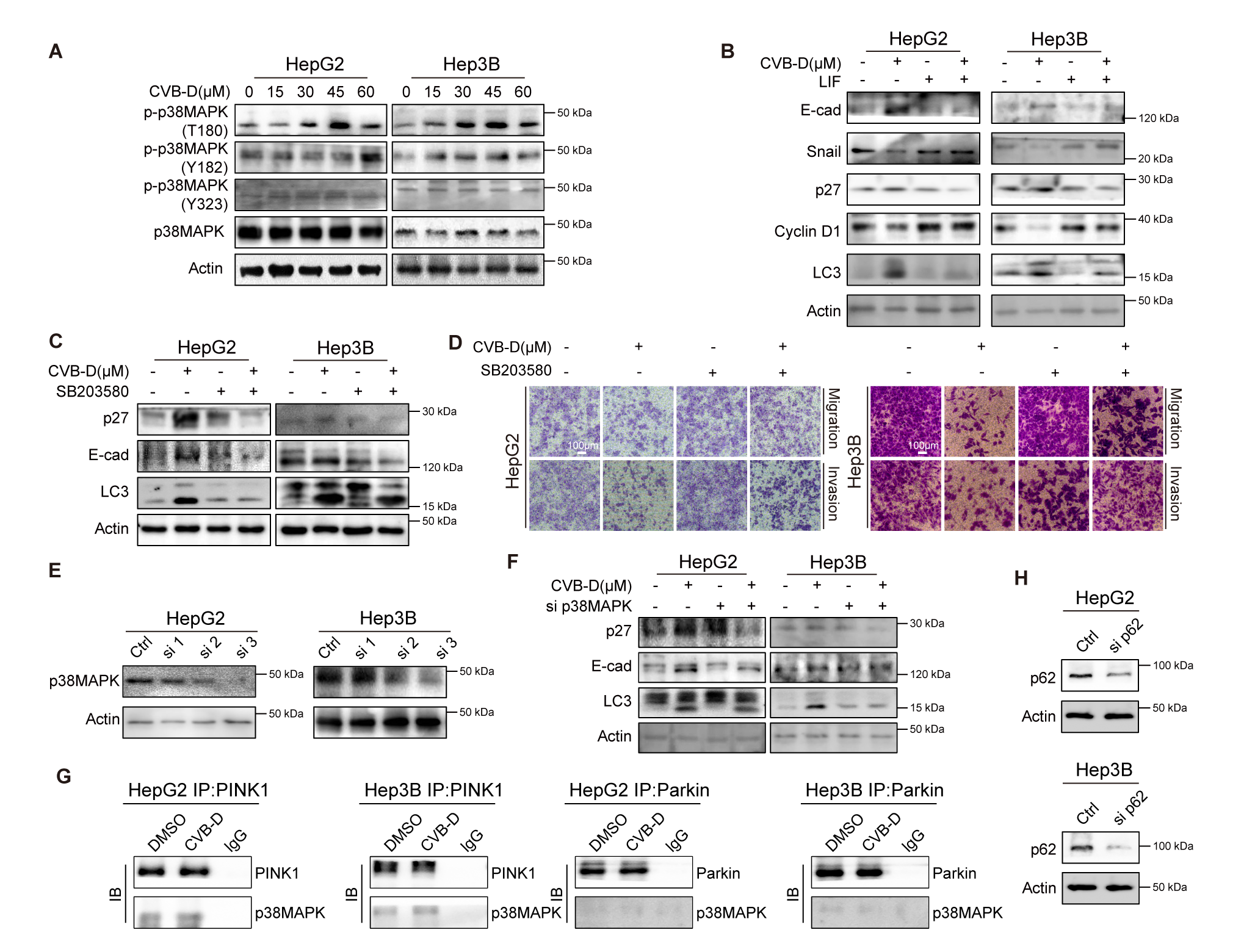
Figure S5.

**Figure S5. CVB-D activates p38MAPK/p62 in HCC via inhibiting LIF** (related to **Fig.5**)**.** (**A**) Phosphorylation levels of p38MAPK were increased by CVB-D. (**B**) Western blotting of E-cad, Snail, p27, Cyclin D1 and LC3 in HepG2 and Hep3B cells that had been treated with or without CVB-D for LIF-overexpression in HCC cells. (**C**) Expression levels of p27, E-cad and LC3 that had been treated with or without CVB-D for adding SB203580. (**D**) Effects of SB203580 on cell migratory and invasive capacities by transwell assays in HCC cells. Scale bar, 100 μm. (**E**) The knockdown efficiency of p38MAPK in HCC cells verified by western blotting. (**F**) Expression levels of p27, E-cad and LC3 that had been treated with or without CVB-D for transferring siRNA of p38MAPK. (**G**) PINK1 and Parkin protein interaction with p38MAPK protein was inhibited by CVB-D. (**H**) Western blotting verified that the siRNA of p62.

Figure S6.


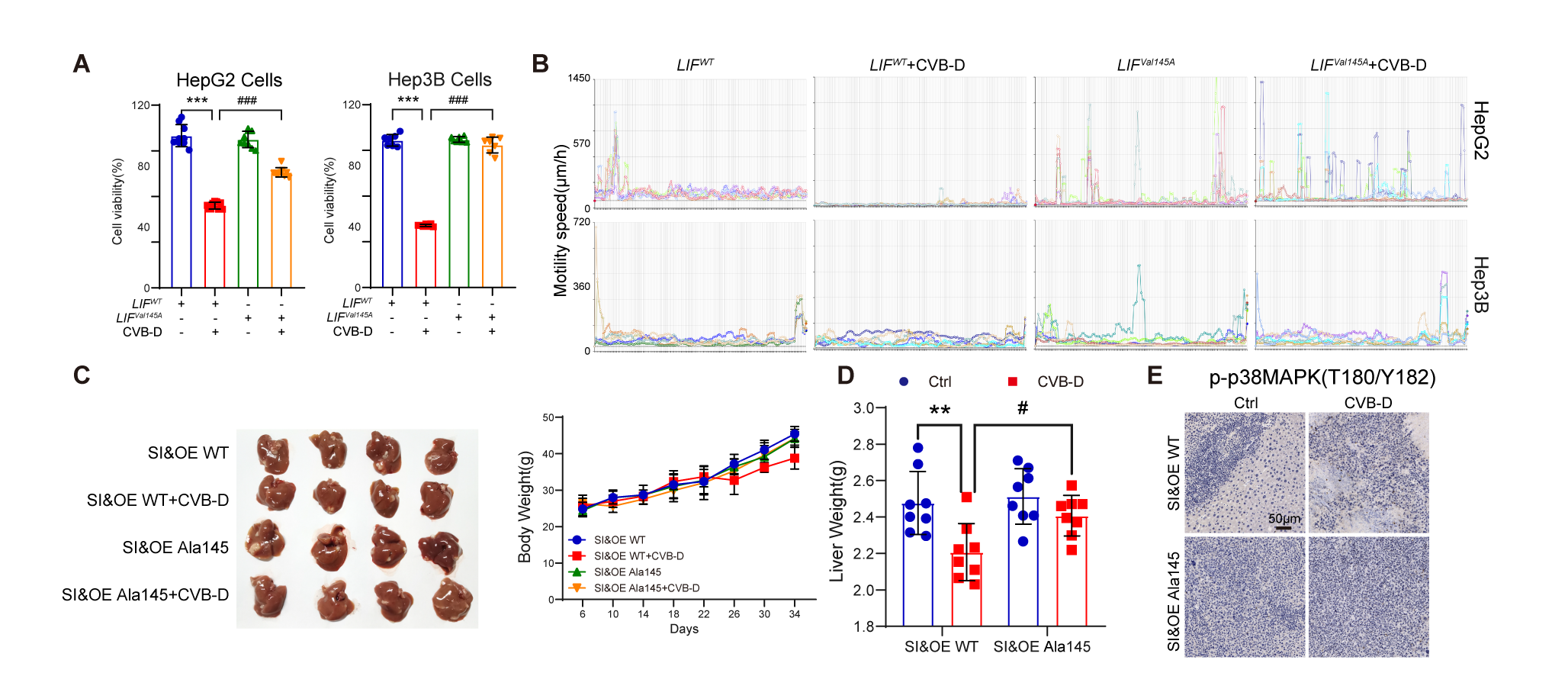


**Figure S6. Inhibition of hepatocarcinogenesis by CVB-D targeted LIF is facilitated by Valine 145** (related to **Fig.6**). **(A)** MTT assays showed CVB-D inhibited cell growth in wt-LIF HCC cell but not LIF-Val145 mutant. **(B)** The HCC cells transfected with vector wt-LIF or LIF-Val145 mutant were analyzed for single-cell motility with quantification of speed of cell movements. **(C, D)** The weight of tumors formed and body weight in orthotopic mice was measured and compared (n = 8 per group). **(E)** Immunohistochemical staining of phosphorylation levels of p38MAPK in tumor tissues. Scale bar, 50 μm. ***p* < 0.01; ****p* < 0.001; *^#^p* < 0.05; *^###^p* < 0.001. Data represent mean ± SEM.

Figure S7.


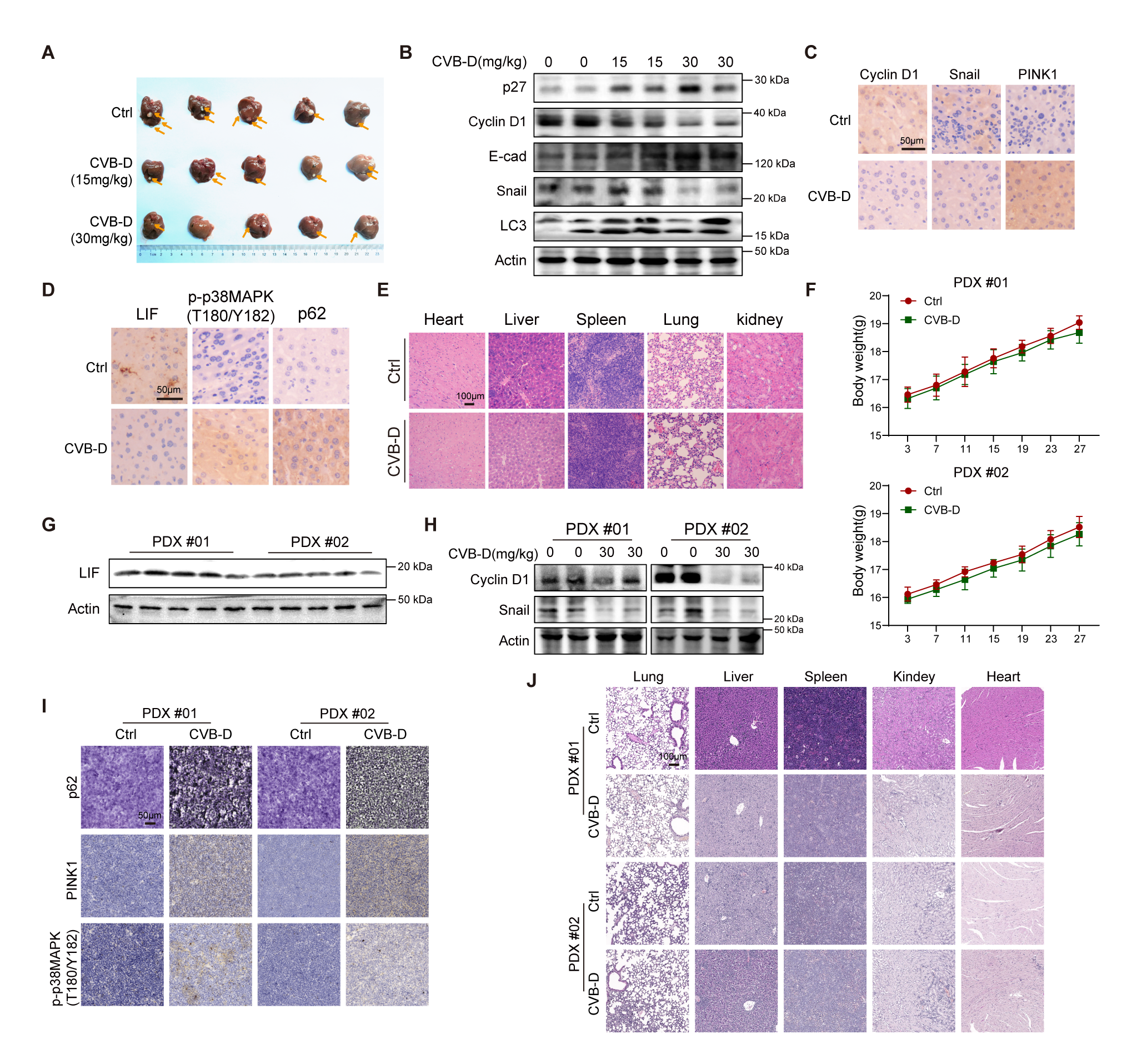


**Figure S7. CVB-D inhibits HCC progression in orthotopic mice and PDX mice** (related to **Fig.7**)**.** (**A**) CVB-D was administered to mice bearing orthotopic tumors by i.p. injection at a dose of 15 mg/kg, 30 mg/kg for once every two days, respectively. (**B**) Expression levels of p27, LC3, Cyclin D1 and Snail were inhibited increased, and the expression levels of E-cad was increased by CVB-D. (**C, D**) Representative staining of LIF, p-p38MAPK(Thr180/Tyr182), Cyclin D1, Snail, p62 and PINK1 in HCC tissues by immunohistochemical. Scale bar, 50 μm. (**E**) Representative hematoxylin and eosin (HE) staining images indicate the effects of CVB-D from the orthotopic mice model. Scale bar, 100 μm. (**F**). Body weight in the two groups is presented. (**G**) Expression of LIF in the PDX from liver cancers of two patients by western blotting. (**H**) Changes in Cyclin D1 and Snail by CVB-D treatment detected using western blotting of HCC tissues from the PDX model. (**I**) Immunohistochemical staining of p62, PINK1 and p-p-38MAPK in tumor tissues. Scale bar, 50 μm. (**J**) Representative HE staining images indicate the effects of CVB-D from the PDX model. Scale bar, 100 μm.

Table S1.

| **Case No.** | | **Gender** | **Tumor Grade** | | **Tumor size (cm)** | **carcinoembryonic antigen (CEA) (ng/ml)** | **Alpha fetoprotein (AFP)** |
| --- | --- | --- | --- | --- | --- | --- | --- |
| #1 | Male | | Ⅱ | 3.9 ×3.3 | | 1.4 | 508.4 |
| #2 | Female | | Ⅱ-Ⅲ | 2.5 ×2 | | 2 | 1.7 |
| #3 | Male | | Ⅰ-Ⅱ | 11 ×6 ×5.5 | | 2.3 | 11.2 |
| #4 | Male | | Ⅱ | 1 ×0.8 | | 1.6 | 15 |
| #5 | Male | | Ⅰ-Ⅱ | 3 ×2 | | 3.7 | 4.5 |
| #6 | Female | | Ⅱ | 3.5 ×3 | | 0.9 | 6179.7 |
| #7 | Male | | Ⅱ | 3.5 ×2 | | 3.6 | 6.8 |
| #8 | Male | | Ⅰ-Ⅱ | 5.5 ×4 | | 1.6 | 14.3 |
| #9 | Female | | Ⅱ | 2 ×1.5 | | 1.8 | 284.3 |
| #10 | Male | | Ⅱ | 5 ×4 ×5 | | 2.7 | 16.2 |

**Table S1.** The details of the HCC patients used in this study are summarized in Table S1.

Table S2.

| **Case No.** | PDX#01 | PDX#02 |
| --- | --- | --- |
| Gender | Male | Female |
| Pathological diagnosis | Hepatocellular carcinoma | Hepatocellular carcinoma |
| Tumor size (cm) | 2.5 ×2.2 ×2.2 | 1.7×1.5×1.4 |
| Tumor stage | Ⅲ | Ⅱ |

**Table S2.** Patient (PDX model) information was shown in Table S2.

**Table S3.**

| si LIF | si#1 | 5’-CAACAACCUGGACAAGCUAUGUGGC-3’ |
| --- | --- | --- |
|  | si#2 | 5’-GUCACAACCUCAUGAACCAGAT-3’ |
| si p38MAPK | si#1 | 5’-GGGAGGUGCCCGAACGAUA-3’ |
|  | si#2 | 5’-GGUCUGUUGGAUGUGUUCA-3’ |
|  | si#3 | 5’-CGACGACCACGUUCAGUUU-3’ |
| si p62 | | 5’-UACAAAUUUAACAGGAUGG-3’ |
| overexpression LIF | | 5’-CGCAAATGGGCGGTAGGCGTG-3’ |

**Table S3.** siRNA and overexpression RNA information was shown in Table S3.

Table S4.

| **Antibodies** | **SOURCE** | **IDENTIFIER** | **Antibody dilutions (for WB)** | **Antibody dilutions (for IF)** | **Antibody dilutions (for IHC)** |
| --- | --- | --- | --- | --- | --- |
| p62 | CST | Cat# 8025S | 1:1000 | 1:200 | 1:200 |
| LC3B | CST | Cat# 3868S | 1:1000 | 1:200 | - |
| p38MAPK | CST | Cat# 8690S | 1:1000 | 1:200 | - |
| E-cadherin | CST | Cat# 3195S | 1:1000 | - | - |
| N-cadherin | CST | Cat# 13116S | 1:1000 | - | - |
| Vimentin | CST | Cat# 5741S | 1:1000 | 1:250 | - |
| Cyclin D1 | CST | Cat# 55506S | 1:1000 | 1:200 | 1:200 |
| Actin | CST | Cat# 4970S | 1:1000 | - | - |
| Snail | Abcam | Cat# ab216347 | 1:1000 | 1:250 | 1:250 |
| p-p38MAPK  (T180/Y182) | CST | Cat# 4511 | 1:1000 | 1:250 | 1:250 |
| p-p38MAPK  (T180) | Affinity Biosciences | Cat# AF3457 | 1:800 | - | - |
| p -p38MAPK  (Y182) | Affinity Biosciences | Cat# AF3455 | 1:800 | - | - |
| p -p38MAPK  (Y323) | Affinity Biosciences | Cat# AF3456 | 1:800 | - | - |
| LIF | Affinity Biosciences | Cat# DF13730 | 1:800 | 1:250 | 1:250 |
| PINK1 | Proteintech | Cat# 23274-1-AP | 1:1000 | - | 1:250 |
| Parkin | Proteintech | Cat# 14060-1-AP | 1:1000 | - | - |
| TOM20 | Proteintech | Cat# 11802-1-AP | 1:800 | - | - |
| p27 | Proteintech | Cat# 25614-1-AP | 1:1000 | - | - |
| Ki67 | Abcam | Cat# ab15580 | - | - | 1:250 |

**Table S4.** Antibodies information was shown in Table S4.
